# Phototoxicity avoidance is a potential therapeutic approach for retinal dystrophy caused by EYS dysfunction

**DOI:** 10.1172/jci.insight.174179

**Published:** 2024-04-22

**Authors:** Yuki Otsuka, Keiko Imamura, Akio Oishi, Kazuhide Asakawa, Takayuki Kondo, Risako Nakai, Mika Suga, Ikuyo Inoue, Yukako Sagara, Kayoko Tsukita, Kaori Teranaka, Yu Nishimura, Akira Watanabe, Kazuhiro Umeyama, Nanako Okushima, Kohnosuke Mitani, Hiroshi Nagashima, Koichi Kawakami, Keiko Muguruma, Akitaka Tsujikawa, Haruhisa Inoue

**Affiliations:** 1 iPSC-based Drug discovery and Development Team, RIKEN BioResource Research Center, Kyoto, Japan.; 2 Center for iPS Cell Research and Application (CiRA), Kyoto University, Kyoto, Japan.; 3 Department of Ophthalmology and Visual Sciences, Kyoto University Graduate School of Medicine, Kyoto, Japan.; 4 RIKEN Center for Advanced Intelligence Project (AIP), Kyoto, Japan.; 5 Department of Ophthalmology and Visual Sciences, Nagasaki University, Nagasaki, Japan.; 6 Division of Molecular and Developmental Biology, National Institute of Genetics, Mishima, Japan.; 7 Graduate School of Medicine, Kyoto University, Kyoto, Japan.; 8 Meiji University International Institute for Bio-Resource Research, Kawasaki, Japan.; 9 Division of Systems Medicine and Gene Therapy, Faculty of Medicine, Saitama Medical University, Saitama, Japan.; 10 Department of iPS Cell Applied Medicine, Graduate School of Medicine, Kansai Medical University, Hirakata, Osaka, Japan.

**Keywords:** Ophthalmology, Stem cells, Retinopathy

## Abstract

Inherited retinal dystrophies (IRDs) are progressive diseases leading to vision loss. Mutation in the eyes shut homolog (*EYS*) gene is one of the most frequent causes of IRD. However, the mechanism of photoreceptor cell degeneration by mutant EYS has not been fully elucidated. Here, we generated retinal organoids from induced pluripotent stem cells (iPSCs) derived from patients with *EYS*-associated retinal dystrophy (*EYS*-RD). In photoreceptor cells of RD organoids, both EYS and G protein–coupled receptor kinase 7 (GRK7), one of the proteins handling phototoxicity, were not in the outer segment, where they are physiologically present. Furthermore, photoreceptor cells in RD organoids were vulnerable to light stimuli, and especially to blue light. Mislocalization of GRK7, which was also observed in *eys*-knockout zebrafish, was reversed by delivering control EYS into photoreceptor cells of RD organoids. These findings suggest that avoiding phototoxicity would be a potential therapeutic approach for *EYS*-RD.

## Abstract 2

Human-dereved organoids of retinal disease, which cannot be modeled in rodents, provide insight into the potential of light-avoidance therapies.

## Graphical abstract

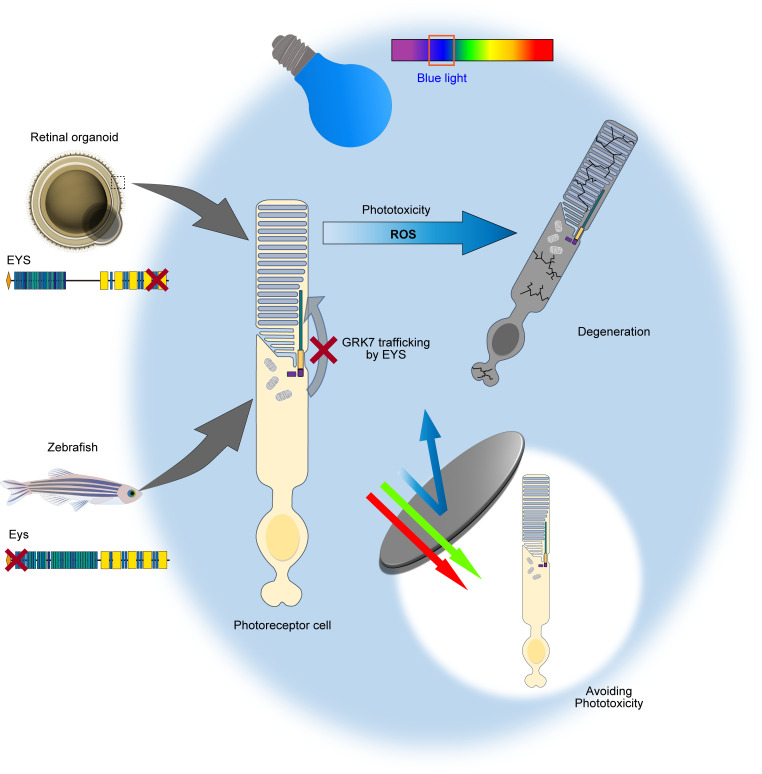

## Introduction

Inherited retinal dystrophies (IRDs) are characterized by progressive photoreceptor cell death that can lead to irreversible vision loss. They are a major cause of visual disability and blindness, affecting more than 4.5 million people worldwide (1). Despite significant progress during the past decade, treatment for IRD is extremely difficult and there are still many challenges to overcome. To date, variants in more than 250 genes have been identified to cause IRD (Retinal Information Network [RetNet], https://sph.uth.edu/retnet/).

The eyes shut homolog (*EYS*) gene was first identified as the autosomal recessive causative gene for retinitis pigmentosa (RP) in 2008 (2). *EYS* is predominantly expressed in the retina and especially in photoreceptor cells (Human Protein Atlas; https://www.proteinatlas.org/ENSG00000188107-EYS/tissue). Variants in the *EYS* gene have been reported to be one of the most frequent RP causes, with a prevalence of approximately 5%–30% (3–8). *EYS* is located on chromosome 6p12, spanning over 2 Mb of genomic DNA. The full-length human *EYS* transcript is composed of 44 exons and encodes the 3,165–amino acid protein that consists of 5 laminin A G–like domains and 28 epidermal growth factor (EGF) or EGF-like domains (2, 3). EYS is one of the largest molecules expressed in the human eye, and its size has resulted in severe limitations in both basic research and gene therapy (9).

*EYS* is missing or interrupted in the genome of several mammalian lineages, including the most common animal models: mouse, rat, and guinea pig (2), and this has made it difficult to investigate *EYS*-associated retinal dystrophies (*EYS*-RDs). Thus, zebrafish have been used as an alternative vertebrate model to study the role of the Eys protein. According to these studies, loss of function is thought to be the molecular mechanism underlying *EYS*-RD (10–13), and Eys is expected to be involved in the maintenance of the morphological structure (10) of photoreceptors or protein transport to outer segments (OSs) (14). Meanwhile, the positions of variants tend to affect the phenotype and disease severity in patients with *EYS*-RD (15), suggesting that additional mechanisms may exist. Although there is 1 report that describes the histopathology of *EYS*-RD in 2 families (c.2259+1G>A and c.2620C>T in family 1, and c.4350_4356del and c.2739_3244del in family 2) (16), the investigated cases were in the advanced stages of the disease and the photoreceptor layer had already become highly disorganized. Therefore, the exact molecular function of EYS and how mutant EYS causes retinal degeneration are yet to be elucidated.

It is essential to exploit human samples for a better understanding of EYS function and involvement of mutant EYS in the pathogenesis of IRDs. Human induced pluripotent stem cells (iPSCs) have enabled us to generate human organ–specific cells and in vitro models to study various diseases (17). Regarding the retina, methods to produce 3-dimensional retinal organoids have been developed (18, 19) and used for the modeling of several retinal diseases, including glaucoma (20), Leber’s congenital amaurosis (21), Stargardt disease (22), and X-linked juvenile retinoschisis (23). In particular, the pathogenesis of several RP-causative genes such as *RP2* (24), *RPGR* (25), and *USH2A* (26) has been investigated with patient-derived organoids, recapitulating the disease phenotypes. These studies have demonstrated that human iPSCs provide a promising model for the study of IRD-associated molecular pathogenesis.

To investigate the pathogenesis of *EYS*-RD, retinal organoids generated from patient-derived iPSCs were analyzed. This is the first report to our knowledge about human iPSCs being used to study the molecular mechanism of EYS function and its pathogenesis. In the current study, human EYS was localized at the connecting cilia (CC) and OS in photoreceptor cells, whereas mutant truncated EYS did not distribute to these regions. We found that EYS plays a role in the trafficking of an OS protein, G protein–coupled receptor kinase 7 (GRK7), which is evolutionarily conserved along with EYS and involved in photoresponse recovery (27, 28). *EYS*-RD organoids and zebrafish exhibited light-induced photoreceptor cell death related to GRK7 deprivation in the OS. Furthermore, light-induced cell death in RD organoids was especially caused by blue light.

## Results

### Generation of retinal organoids from EYS-RD patient iPSCs.

In the current study, we generated iPSCs from 2 patients with *EYS*-RD (Supplemental Table 1; supplemental material available online with this article; https://doi.org/10.1172/jci.insight.174179DS1). Clinical phenotypes of these patients, including fundus photographs and spectral domain optical coherence tomography (SD-OCT) images, are shown in Supplemental Figure 1A. In RD patient 1, the macular region had already degenerated, with almost no central vision. Moreover, there was subtle cone function in electroretinography (ERG) and central and temporal visual fields were spared in RD patient 2. Two healthy iPSCs established in a previous study (29) were used as a control. All iPSC clones expressed pluripotent markers upon immunostaining (Supplemental Figure 1B). Both patients, who were sporadic and from independent families, had homozygous c.8805C>A (p.Y2935X) variants in the *EYS* gene (Supplemental Figure 1C). This is one of the most frequent variants in the Japanese population (30, 31), and they showed normal karyotypes (Supplemental Figure 1D).

**Figure 1:**
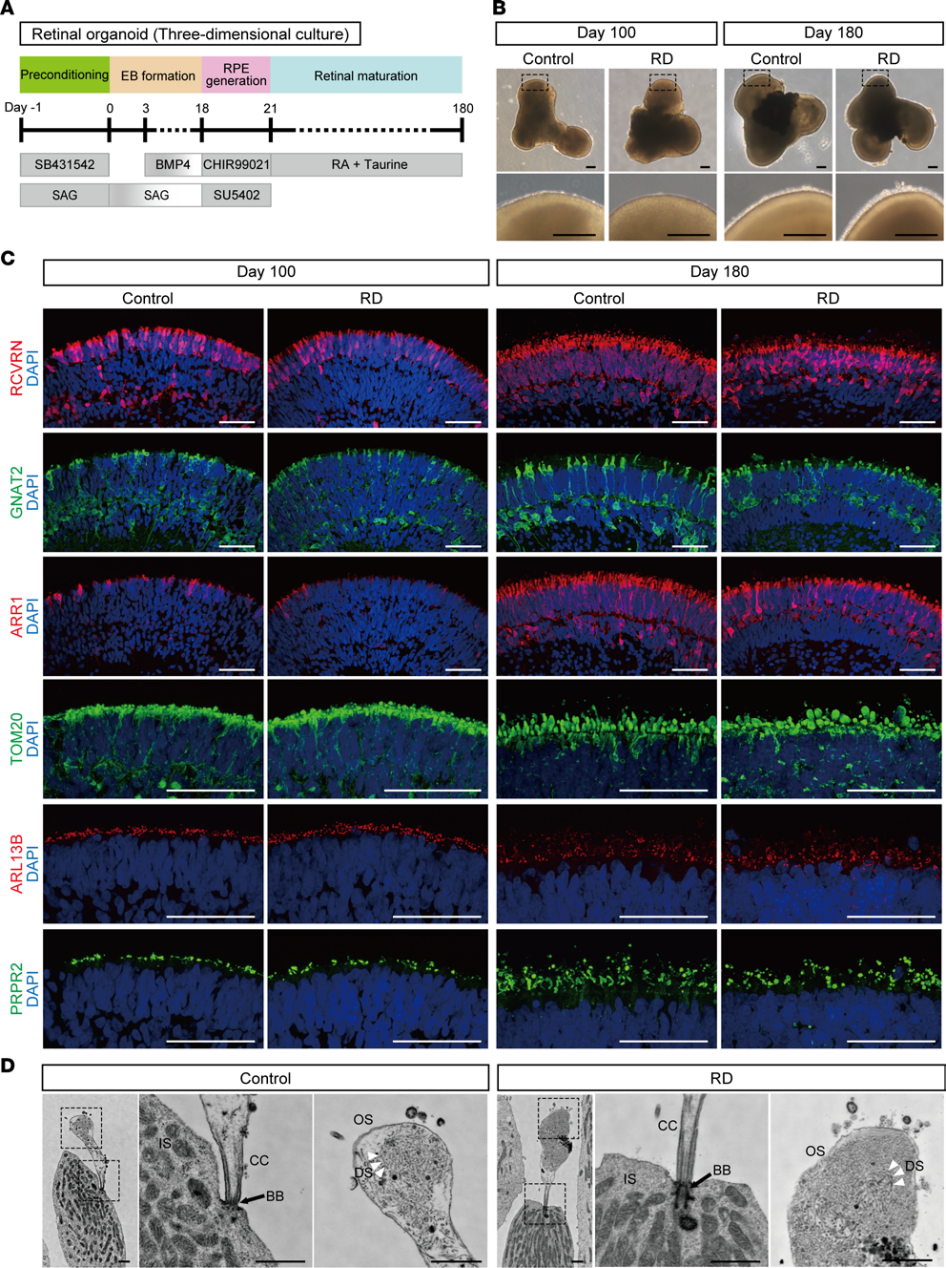
Generation of retinal organoids from human iPSCs. (**A**) Differentiation protocol to generate retinal organoids. Feeder-free human iPSCs were preconditioned 1 day before the differentiation. Serum-free floating culture of embryoid body–like (EB-like) aggregates with the quick aggregation (SFEBq) method (see Methods) was employed. SAG, smoothened agonist; RA, retinoic acid; RPE, retinal pigment epithelium. (**B**) Bright-field view of differentiated retinal organoids on day 100 and day 180. Lower panels are higher-magnification images of the dotted boxes in the upper panels. Scale bars: 200 μm. (**C**) Representative immunofluorescence images of retinal organoids on day 100 and day 180 stained for the panphotoreceptor marker recoverin (RCVRN), rod cell marker arrestin 1 (ARR1), cone cell marker G protein subunit α transducin 2 (GNAT2), mitochondrial marker translocase of outer mitochondrial membrane 20 (TOM20), cilia marker ADP ribosylation factor–like GTPase 13B (ARL13B), and outer segment marker peripherin 2 (PRPH2). Scale bars: 50 μm. (**D**) Scanning electron microscopy images of photoreceptor cells in retinal organoids on day 180. Middle and right panels are higher-magnification images of the dotted boxes in the left panels. BB, basal body; CC, connecting cilium; DS, disc stack; IS, inner segment; OS, outer segment. Scale bars: 1 μm.

Retinal organoids were generated from control and RD iPSCs according to a previously described protocol (32) (Figure 1A). To investigate the cellular constituents of organoids, single-cell RNA sequencing was conducted on day 70 (Supplemental Figure 2A). The generation of diverse retinal cells was confirmed by their gene expression of cell type–specific markers (Supplemental Figure 2, B and C). Photoreceptor cells exhibited EYS expression (Supplemental Figure 2D), the levels of which were not different between control and RD (Supplemental Figure 2E).

In organoids, a transparent laminated neural retinal layer was generated by day 100, and then brush-like borders developed on the surface by day 180 (Figure 1B). The generation of photoreceptor cells was confirmed by the expression of recoverin (RCVRN), G protein subunit α transducin 2 (GNAT2), and S-antigen visual arrestin (arrestin 1, ARR1) by immunostaining on day 100 and day 180. RCVRN staining showed that the photoreceptor cell layer was well formed on the surface of organoids. The expression of GNAT2 and ARR1, specific markers of cone and rod cells, respectively, was enhanced in both groups, depending on the culture period. More specifically, immunostaining for mitochondrial marker translocase of outer mitochondrial membrane 20 (TOM20), ciliary marker ADP ribosylation factor–like GTPase 13B (ARL13B), and OS marker peripherin 2 (PRPH2) revealed that divided compartments representing inner segment (IS), CC, and OS structures were produced in the apical side of the photoreceptor cell (Figure 1C). The staining pattern and amount of mature rod cell marker rhodopsin were not different (Supplemental Figure 3, A and B), suggesting that the differentiation status was similar between control and RD organoids used in this study. In addition, intraflagellar transport protein 88 (IFT88), a marker of IFT complex component B, localized at the proximal region of OSs stained for PRPH2, showing that anterograde ciliary transport had developed (Supplemental Figure 3C). The robust ultrastructure, including IS, CC, and nascent OS with disc stacks had developed in photoreceptor cells of both control and RD, as determined by scanning electron microscopy (Figure 1D).

### EYS was mislocalized in photoreceptor cells of RD retinal organoids.

As there have been no reports to our knowledge describing EYS immunostaining of human retinal organoids, we first confirmed the validity of commercially available anti-EYS antibodies. We generated plasmid vectors containing full-length EYS or c.8805-truncated EYS, with GFP separated by internal ribosomal entry site (IRES) sequences (WT-EYS-GFP and MT-EYS-GFP, respectively) (Supplemental Figure 4A). These vectors were transfected into 293T cells and GFP fluorescence was observed (Supplemental Figure 4B). By Western blot analysis, anti-EYS antibodies from Atlas Antibodies and Creative Diagnostics were demonstrated to detect EYS protein. It is of note that an approximately 325 kDa mutant EYS was also recognized and detected by these antibodies (Supplemental Figure 4C). Furthermore, we confirmed that robust EYS staining was observed in some EYS-overexpressing 293T cells and pig retina with the anti-EYS antibody from Atlas Antibodies (Supplemental Figure 4, D and E). These results demonstrate that the Atlas antibody is suitable for detecting human EYS by both Western blot analysis and immunocytochemistry, and so we decided to use it for further experiments.

c.8805C>A (p.Y2935X) is a nonsense mutation located in the last EGF domain near the C-terminus (Supplemental Figure 1C), producing truncated EYS protein rather than causing nonsense-mediated decay (NMD), based on a previous report (33). Because of the position of c.8805C>A near the C-terminus, we considered the possibility that mutant EYS may contribute a gain-of-toxic-function mechanism. 293T cells overexpressing GFP, control EYS, or mutant EYS were evaluated by Western blotting with the apoptosis marker cleaved caspase-3 and ER stress markers such as binding immunoglobulin protein (BiP), eukaryotic initiation factor 2α (eIF2α), phospho-eIF2α (p-eIF2α), PKR-like ER kinase (PERK), p-PERK, and activating transcription factor 6 (ATF6). Cleaved caspase-3 was not detected (data not shown), and the amount of ER stress markers was not different between control and mutant EYS–overexpressing cells (Supplemental Figure 4, F–J).

**Figure 2:**
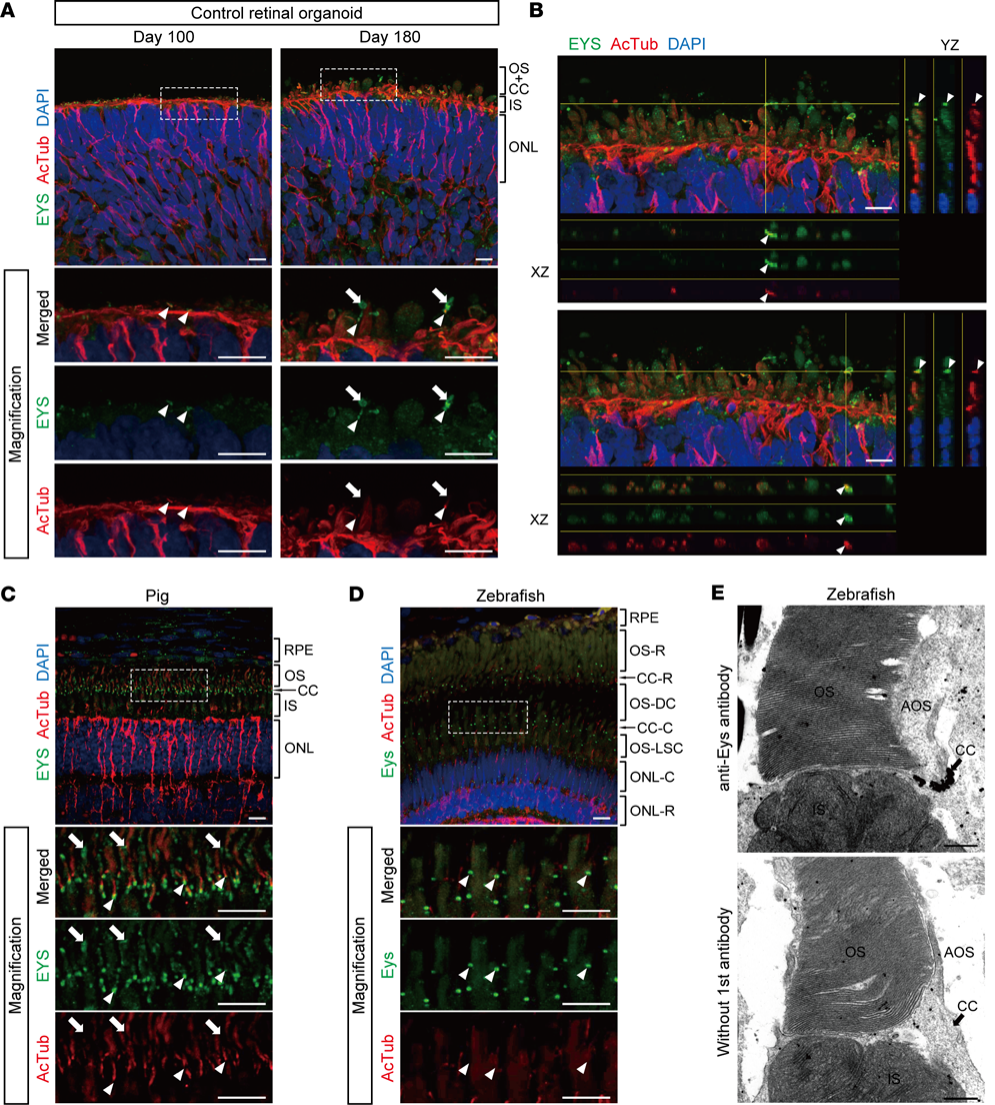
EYS localization in human retinal organoids, pig, and zebrafish. (**A**) Representative immunofluorescence images of control retinal organoids on day 100 and day 180 stained for EYS and acetylated α-tubulin (AcTub). Lower panels are higher-magnification images of the dotted boxes in the upper panels. White arrowheads indicate the CC, and white arrows indicate the nascent OS. ONL, outer nuclear layer. Scale bars: 10 μm. (**B**) Two representative immunostaining images of control retinal organoids on day 180, using orthogonal projections. EYS immunoreactivity colocalized with AcTub (white arrowheads). Scale bars: 10 μm. (**C**) Representative immunohistochemistry images of EYS and AcTub in WT pig retina. Lower panels are higher-magnification images of the dotted box in the upper panel. White arrowheads indicate the CC, and white arrows indicate the OS. RPE, retinal pigment epithelium. Scale bars: 10 μm. (**D**) Representative immunohistochemistry images of Eys and AcTub in WT zebrafish retina. Lower panels are higher-magnification images of the dotted box in the upper panel. White arrowheads indicate the CC. CC-C, cone CC; CC-R, rod CC; ONL-C, cone ONL; ONL-R, rod ONL; OS-DC, double cone outer segment; OS-LSC, long single cone outer segment; OS-R, rod outer segment. Scale bars: 10 μm. (**E**) Representative immunoelectron microscopy images of WT zebrafish retina. Upper panel shows staining with anti-Eys antibody. Negative control without the primary antibody is shown in the lower panel. AOS, accessory outer segment. Scale bars: 500 nm.

Next, we investigated the expression and localization of EYS in control retinal organoids by immunostaining. On day 100, EYS was observed around the organoid surface, dominantly colocalizing with ciliary structures stained with acetylated α-tubulin (Figure 2A and Supplemental Figure 4K). On day 180, intense EYS staining was observed extending from the acetylated α-tubulin–positive region to the apical side (Figure 2A and Supplemental Figure 4K). *Z*-stack images with orthogonal projections revealed that EYS colocalized with acetylated α-tubulin in retinal organoids (Figure 2B). Furthermore, we extended the culture period to day 300, and confirmed that EYS staining was observed throughout the developed OS (Supplemental Figure 4L). These results suggested that EYS was localized to the ciliary region distributing to nascent OS, as a photoreceptor ultrastructure had developed.

We also evaluated EYS localization in the retina of WT pigs and zebrafish. In pig retina, EYS staining was detected not just at the basal part of acetylated α-tubulin but also the OS (Figure 2C and Supplemental Figure 4K). Furthermore, EYS staining exhibited a punctate pattern and localized only at the basal end of acetylated α-tubulin in zebrafish (Figure 2D and Supplemental Figure 4K), as described previously (10, 34, 35). Immunoelectron microscopy with WT zebrafish also confirmed the intense EYS staining at the CC, although other localization, including the OS, was not obvious (Figure 2E). These staining patterns were consistent with our findings using human retinal organoids.

**Figure 3:**
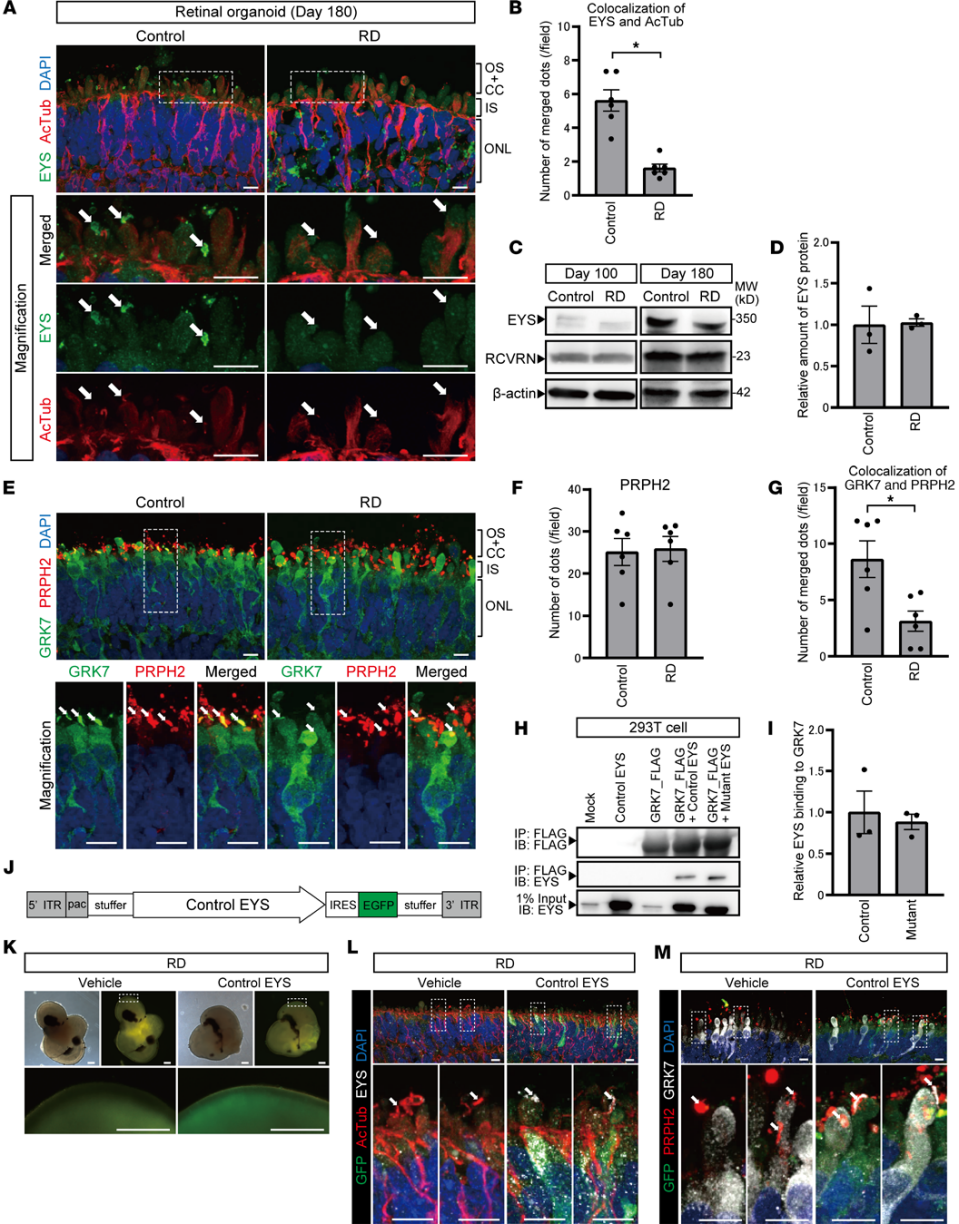
RD retinal organoids exhibited mislocalization of EYS and GRK7 in connecting cilia and outer segment. (**A**) Representative immunofluorescence images of EYS and acetylated α-tubulin (AcTub) in control and RD retinal organoids on day 180. Lower panels are higher-magnification images of the dotted boxes in the upper panels. White arrows indicate the CC and nascent OS. Scale bars: 10 μm. (**B**) Quantitative analysis of merged immunoreactivity of EYS and AcTub in control and RD retinal organoids. The *y* axis indicates the number of merged dots per field. Data represent mean ± SEM (*n* = 6 organoids). An unpaired, 2-tailed *t* test was used for statistical comparison (*P < 0.05). (**C**) Western blot analysis of EYS and RCVRN in control and RD retinal organoids on day 100 and day 180. Full-length pictures of the blots are presented in Supplemental Figure 8, A–F. (**D**) Quantification of Western blot bands in **C**. The *y* axis indicates the relative amount of intracellular EYS protein. Data represent mean ± SEM from independent experiments (*n* = 3). An unpaired, 2-tailed *t* test was used for statistical comparison (*P* = 0.92). (**E**) Representative immunofluorescence images of GRK7 and OS marker PRPH2 in control and RD retinal organoids on day 180. Lower panels are higher-magnification images of the dotted boxes in the upper panels. White arrows indicate the OS region. Scale bars: 10 μm. (**F**) Quantitative analysis of PRPH2 immunoreactivity in control and RD retinal organoids in **E**. The *y* axis indicates the number of reactive dots per field. Data represent mean ± SEM (*n* = 6 organoids). An unpaired, 2-tailed *t* test was used for statistical comparison (*P* = 0.87). (**G**) Quantitative analysis of merged immunoreactivity of PRPH2 and GRK7 in control and RD retinal organoids in **E**. The *y* axis indicates the number of reactive dots per field. Data represent mean ± SEM (*n* = 6 organoids). An unpaired, 2-tailed *t* test was used for statistical comparison (*P < 0.05). (**H**) Biochemical interactions between EYS and GRK7. 293T cell lysates were subjected to immunoprecipitation (IP) using anti-FLAG antibody and immunoblotted (IB) with antibodies against FLAG (top) or EYS (middle and bottom). A full-length picture of the blot is presented in Supplemental Figure 9. (**I**) Quantification of IB bands in **H**. The *y* axis indicates the relative amount of control or mutant EYS binding to GRK7. Data represent mean ± SEM from independent experiments (*n* = 3). An unpaired, 2-tailed *t* test was used for statistical comparison (*P* = 0.69). (**J**) Schematic of helper-dependent adenoviral vector containing control EYS. IRES, internal ribosome entry site; ITR, inverted terminal repeats; pac, packaging signal; stuffer, stuffer DNA. (**K**) Bright-field and EGFP fluorescence of RD organoids 48 hours after adenoviral infection. Scale bars: 200 μm. (**L**) Representative immunofluorescence images of GFP, EYS, and AcTub in RD retinal organoids after control EYS introduction with adenoviral vector. Lower panels are higher-magnification images of the dotted boxes in the upper panels. White arrows indicate the CC and nascent outer segment OS. Scale bars: 10 μm. (**M**) Representative immunofluorescence images of GFP, GRK7, and PRPH2 in RD retinal organoids after control EYS introduction with adenoviral vector. Lower panels are higher-magnification images of the dotted boxes in the upper panels. White arrows indicate the nascent OS. Scale bars: 10 μm.

Subsequently, we investigated the EYS staining pattern and its localization in RD organoids. EYS staining in cilia or OS regions appeared to be less intense and less frequently observed in RD compared with control (Figure 3A). We analyzed images double-stained for EYS and acetylated α-tubulin and found that their colocalization was significantly disturbed in RD compared with control (Figure 3B). We also confirmed the detection of intracellular EYS protein in RD organoids. By Western blot analysis, both healthy control and mutant EYS were detected in the cell lysate of control and RD organoids, respectively (Figure 3C). The intracellular amount of EYS protein did not significantly differ between control and RD on day 180 (Figure 3D).

### EYS function of transporting GRK7 was impaired in EYS-RD photoreceptor cells.

To identify the OS region of photoreceptor cells in retinal organoids, we used PRPH2 as an OS marker. The OS region stained for PRPH2 was clearly seen around the surface of organoids as bulging of the ciliary plasma membrane (Figure 1C), as previously reported (36). We confirmed that the apparent interaction between PRPH2 and EYS was not detected by immunoprecipitation (IP) (Supplemental Figure 5A), suggesting that PRPH2 trafficking to the OS is independent of EYS.

Among various OS proteins, we hypothesized that human EYS is associated with the trafficking of GRK7 that is involved in the shutoff and recovery of the photoresponse, in that both EYS and GRK7 are expressed more dominantly in cone cells than in rod cells and are evolutionarily lost in some common mammals. To evaluate GRK7 localization in photoreceptor cells, we performed immunostaining using organoids on day 180. In controls, GRK7 was detected in the apical side of cilia and OS-like structures, colocalizing with PRPH2 (Figure 3E). Meanwhile, in RD, although the cytoplasm was well delineated with GRK7 as well as controls, GRK7 localization in the OS was less frequently observed (Figure 3E). We quantified the data and found that the number of OS marker PRPH2 in the OS was not different between control and RD organoids (Figure 3F), indicating that the differentiation status of both organoids was virtually identical. However, the number of merged dots (GRK7 and PRPH2) was significantly greater in controls (Figure 3G)

Subsequently, we examined whether EYS interacts with GRK7 for regulating its transport. For this purpose, 293T cells were transfected with the following 4 combinations of plasmids: (a) control EYS with GFP (Control EYS), (b) GRK7 with FLAG (GRK7_FLAG), (c) Control EYS and GRK7_FLAG, and (d) mutant EYS with GFP (Mutant EYS) and GRK7_FLAG (Supplemental Figure 5B). Protein extracts were used for IP with anti-FLAG antibody and immunoblotted with antibodies against FLAG or EYS. FLAG antibody successfully detected all 3 proteins, including GRK7_FLAG (Figure 3H; top, lanes 3–5). Bands at approximately 350 or 325 kDa, which corresponded to Control EYS or Mutant EYS, respectively, were detected in protein extracts from cotransfected 293T cells (Figure 3H; middle, lanes 4 and 5), but not in other lanes (Figure 3H; middle, lanes 1–3). Quantifying the amount of EYS protein binding to GRK7 showed that there was no significant difference between Control EYS and Mutant EYS (Figure 3I). Meanwhile, we also evaluated the localization and interaction of the other opsin kinase, GRK1. However, the amount of staining in the OS region did not differ between control and RD organoids (Supplemental Figure 5, C and D), and IP analysis revealed no obvious interaction between human EYS and GRK1 (Supplemental Figure 5E). Moreover, we examined the OS localization of other major photoresponse-associated proteins such as RCVRN, GNAT2, and ARR1 in retinal organoids on day 180 (Supplemental Figure 5F). However, there were no significant differences between control and RD (Supplemental Figure 5, G–I).

To rescue the phenotypes identified in RD, we introduced the 9.4-kb cDNA encoding full-length control EYS to RD organoids using an adenoviral vector. A high-capacity helper-dependent adenoviral vector (HDAdV) carrying the control EYS with enhanced GFP (EGFP) (Figure 3J) was used to infect RD retinal organoids on day 200. EGFP fluorescence was observed on the organoid surface, 48 hours after infection (Figure 3K). We found that overexpressed control EYS localized at the CC and OS in EGFP-positive cells (Figure 3L). We also confirmed that GRK7 mislocalization in RD organoids was rescued by replacement with control EYS (Figure 3M).

### GRK7 deprivation from the OS was also detected in EYS-KO retinal organoids.

**Figure 4:**
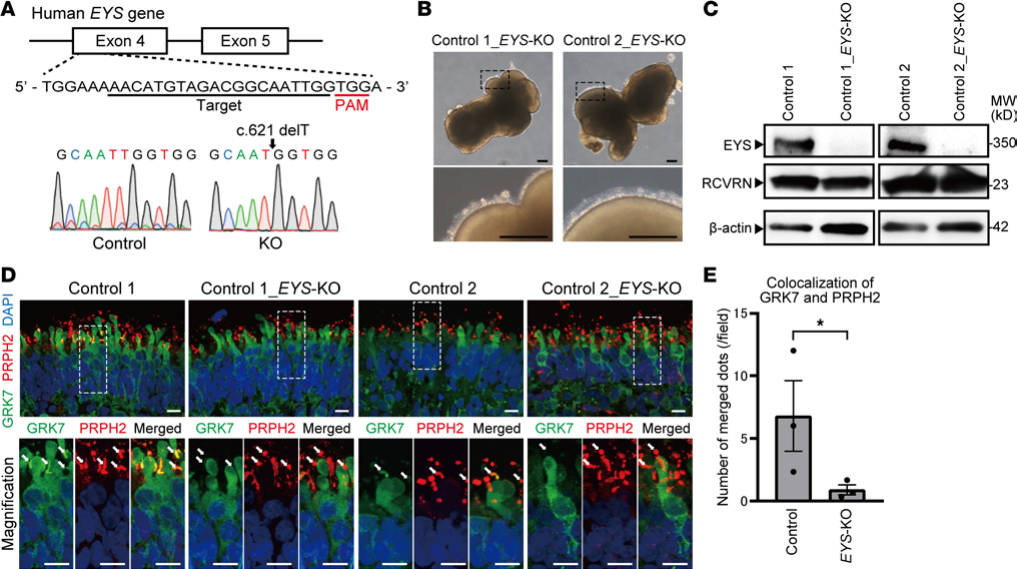
Generation of *EYS*-KO iPSCs with CRISPR/Cas9 system. (**A**) Target sequence of CRSPR/Cas9 gene editing is located in exon 4 of the *EYS* gene. Sanger sequencing of edited iPSCs shows single base deletion (c.621 delT). (**B**) Bright-field view of *EYS*-KO retinal organoids on day 180. Lower panels are higher-magnification images of the dotted boxes in the upper panels. Scale bars: 200 μm. (**C**) Western blot analysis of EYS and RCVRN in control and *EYS*-KO retinal organoids on day 180. Full-length pictures of the blots are presented in Supplemental Figure 8, G–L. (**D**) Representative immunofluorescence images of GRK7 and OS marker PRPH2 in control and *EYS*-KO retinal organoids on day 180. Lower panels are higher-magnification images of the dotted boxes in the upper panels. White arrows indicate the OS region. Scale bars: 10 μm. (**E**) Quantitative analysis of merged immunoreactivity of PRPH2 and GRK7 in control and *EYS*-KO retinal organoids in **D**. The *y* axis indicates the number of reactive dots per field. Data represent mean ± SEM (*n* = 3 organoids). An unpaired, 2-tailed *t* test was used for statistical comparison (*P < 0.05).

To confirm the phenotypes we found with *EYS*-RD retinal organoids, we generated *EYS*-knockout (*EYS*-KO) iPSCs with the CRISPR/Cas9 system. The guides were designed to target the sequences in the fourth exon where the start codon is located (Figure 4A). Two independent iPSC clones with a single base deletion (c.621delT) were successfully generated and expanded for further experiments. This deletion results in a premature termination codon (c.660_662) and is considered to cause NMD (33). Both *EYS*-KO retinal organoids and controls on day 180 showed the development of a transparent laminated neural retinal layer and brush-like borders (Figure 4B). Western blot analysis confirmed the absence of full-length EYS protein in both *EYS*-KO organoids (Figure 4C). GRK7 localization in photoreceptor cells was evaluated by immunostaining of control and *EYS*-KO organoids on day 180. Colocalization of GRK7 and PRPH2 was less frequently detected in *EYS*-KO organoids than in each control (Figure 4D), and there was a significant difference (Figure 4E).

### eys-KO zebrafish also showed Grk7 mislocalization with light-induced photoreceptor cell death.

**Figure 5:**
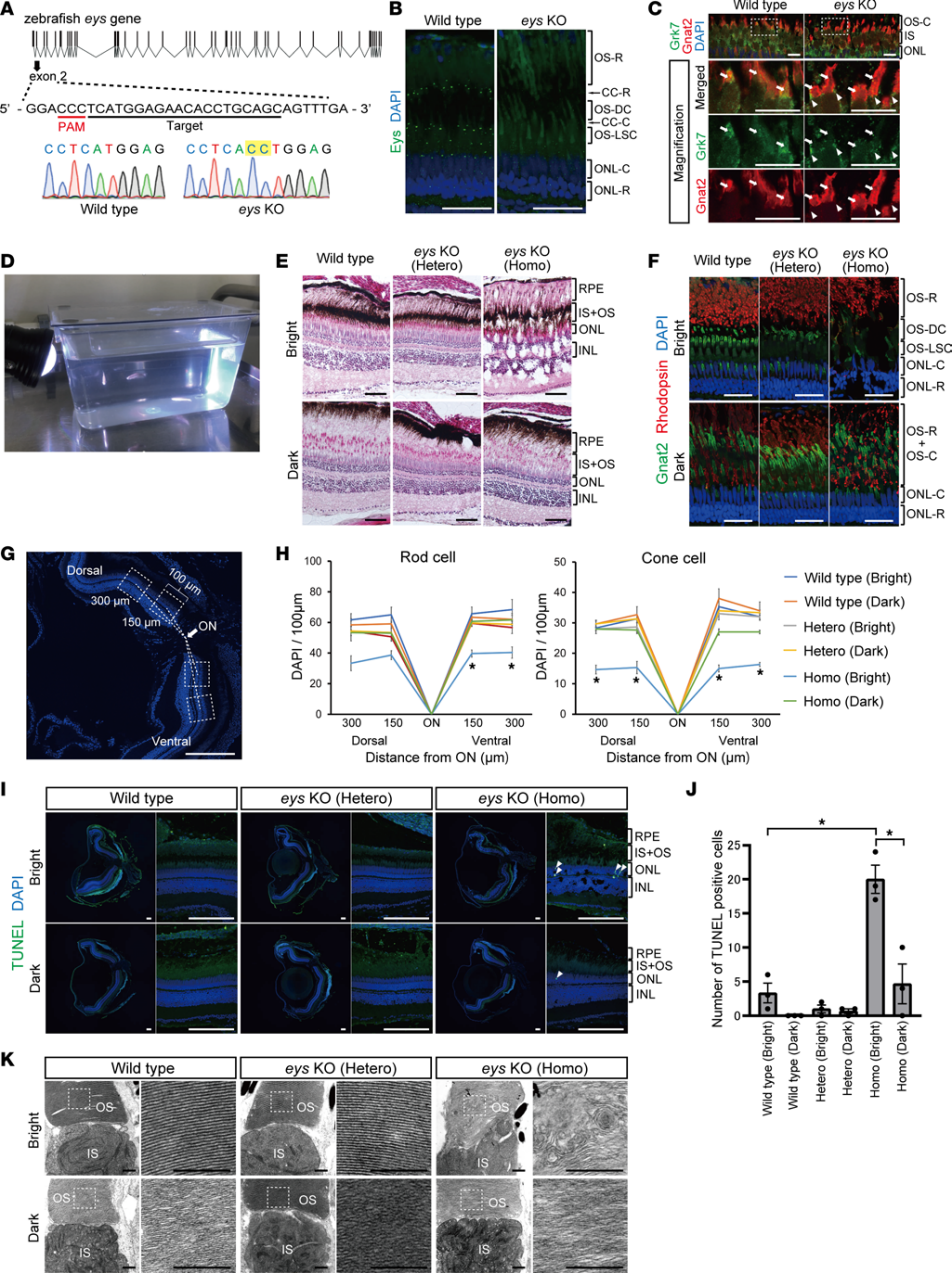
*eys*-KO zebrafish exhibited GRK7 mislocalization in outer segment and light-induced photoreceptor cell death. (**A**) Generation of *eys-*KO zebrafish with CRISPR/Cas9 technology. The zebrafish *eys* genomic structure is shown. The protospacer-adjacent motifs (PAMs) and crRNA targeting sequences in exon 2 are underlined. Sanger sequencing identified a 2–base pair insertion in *eys*-KO zebrafish. (**B**) Representative Eys immunostaining images of WT and *eys*-KO zebrafish. No punctate staining in photoreceptor cells was observed in *eys*-KO zebrafish. CC-C, cone connecting cilium; CC-R, rod connecting cilium; ONL-C, cone outer nuclear layer; ONL-R, rod outer nuclear layer; OS-DC, double cone outer segment; OS-LSC, long single cone outer segment; OS-R, rod outer segment. Scale bars: 20 μm. (**C**) Images of WT and *eys*-KO zebrafish immunostained for Grk7 and OS marker Gnat2. Lower panels are higher-magnification images of the dotted boxes in the upper panels. White arrowheads indicate the CC, and white arrows indicate the OS. OS-C, cone outer segment. Scale bars: 10 μm. (**D**) Three-month-postfertilization WT and *eys*-KO (heterozygous and homozygous) zebrafish were exposed to white LED light. (**E**) Retinal sections of WT and *eys*-KO (heterozygous and homozygous) zebrafish after light stimulation (Bright) or dark adaptation (Dark) were stained with hematoxylin and eosin. Scale bars: 50 μm. (**F**) Representative immunohistochemistry of light-stimulated or dark-adapted WT and *eys*-KO (heterozygous and homozygous) zebrafish. Rhodopsin and Gnat2 identify rod OS and cone OS, respectively. Scale bars: 20 μm. (**G**) Method for counting the number of rod or cone nuclei in light-stimulated or dark-adapted zebrafish. Dotted boxes show areas with a width of 100 μm. Dorsal and ventral areas 150 or 300 μm from the center of optic nerve (ON) were evaluated. Scale bar: 200 μm. (**H**) Quantification of rod or cone cell numbers by nuclei counting in light-stimulated or dark-adapted WT and *eys*-KO (heterozygous and homozygous) zebrafish. The *x* axis indicates the distance from the ON. The *y* axis indicates the number of nuclei within a range of 100 μm. One-way ANOVA with Dunnett’s post hoc test was used for statistical comparison (*P < 0.05). Data represent mean ± SEM (*n* = 3). (**I**) Representative TUNEL staining images of WT and *eys*-KO zebrafish after light stimulation or dark adaptation. White arrowheads indicate TUNEL-positive cells. Scale bars: 100 μm. (**J**) The number of TUNEL-positive cells was quantified in each zebrafish after light exposure or dark adaptation. The *y* axis indicates the number of TUNEL-positive cells per eye. Data represent mean ± SEM (*n* = 3). One-way ANOVA with Dunnett’s post hoc test was used for statistical comparison (*P < 0.05). (**K**) Representative transmission electron microscopy images of photoreceptor cells in WT and *eys*-KO (heterozygous and homozygous) zebrafish. Right panels are higher-magnification images of the OS in the dotted boxes in the left panels. Scale bars: 500 nm.

We generated *eys*-KO zebrafish with a CRISPR/Cas9 system to confirm the phenotype identified in retinal organoids. The sequences in exon 2 were targeted (Figure 5A), and we successfully generated stable zebrafish carrying a 2–base pair insertion (c.619_620insCC; Figure 5A). This is predicted to lead to a frameshift and premature termination of protein translocation (p.Met49Thrfs*8). The total loss of Eys expression in *eys*-KO zebrafish was determined by its absence in immunostaining of the CC (Figure 5B). To evaluate Grk7 localization, we conducted double staining for Grk7 and the cone OS marker, Gnat2. In WT zebrafish, Grk7 staining was observed throughout the cell body, including the OS in cone cells (Figure 5C). Meanwhile, in *eys*-KO zebrafish, Grk7 staining appeared to be limited from the IS to CC, and its transport to the OS was disturbed (Figure 5C). Although photoreceptor cell degeneration had occurred in *eys*-KO zebrafish, other photoreceptor makers, including rhodopsin and Grk1, did not show obvious differences in staining patterns between WT and *eys*-KO zebrafish (Supplemental Figure 6A). These zebrafish phenotypes confirmed our findings in RD and *EYS*-KO organoids.

Subsequently, zebrafish at 3 months postfertilization were stimulated with white LED light before fixation (Figure 5D). Dark-adapted zebrafish were also sacrificed at the same time as a control. Hematoxylin and eosin staining revealed that the retinal structure was remarkably disrupted, with cyst formation only in light-exposed *eys*-KO zebrafish (Figure 5E). Immunohistochemical staining for rod cell (rhodopsin) and cone cell (Gnat2) markers showed different staining patterns between bright and dark states, known as retinomotor movement (37). However, both photoreceptor stainings were drastically reduced in light-exposed *eys*-KO zebrafish, even though there was no obvious difference among dark-adapted fish (Figure 5F). We quantified the number of rod and cone cells by counting nuclei in each zebrafish (Figure 5G and Methods). The numbers of both rod and cone cells were decreased in entire fields of light-stimulated *eys*-KO compared with the other zebrafish, and the photoreceptor cell loss was cone dominant (Figure 5H). To determine whether photoreceptor cell loss in light-stimulated *eys*-KO zebrafish was affected by apoptosis, we carried out TUNEL staining. TUNEL-positive photoreceptor nuclei were most frequently detected in light-stimulated *eys*-KO zebrafish, and there were significant differences from the other zebrafish (Figure 5, I and J). Furthermore, transmission electron microscopy analysis revealed that the arrangement of disc stacks was highly disrupted in light-stimulated *eys-*KO, whereas there were no morphological changes in other samples, including dark-adapted *eys-*KO zebrafish (Figure 5K). Such abnormality in the OS was found in all 3 light-irradiated *eys*-KO zebrafish (Supplemental Figure 6B).

### RD and EYS-KO retinal organoids exhibited light overresponse and light-induced photoreceptor cell death.

**Figure 6:**
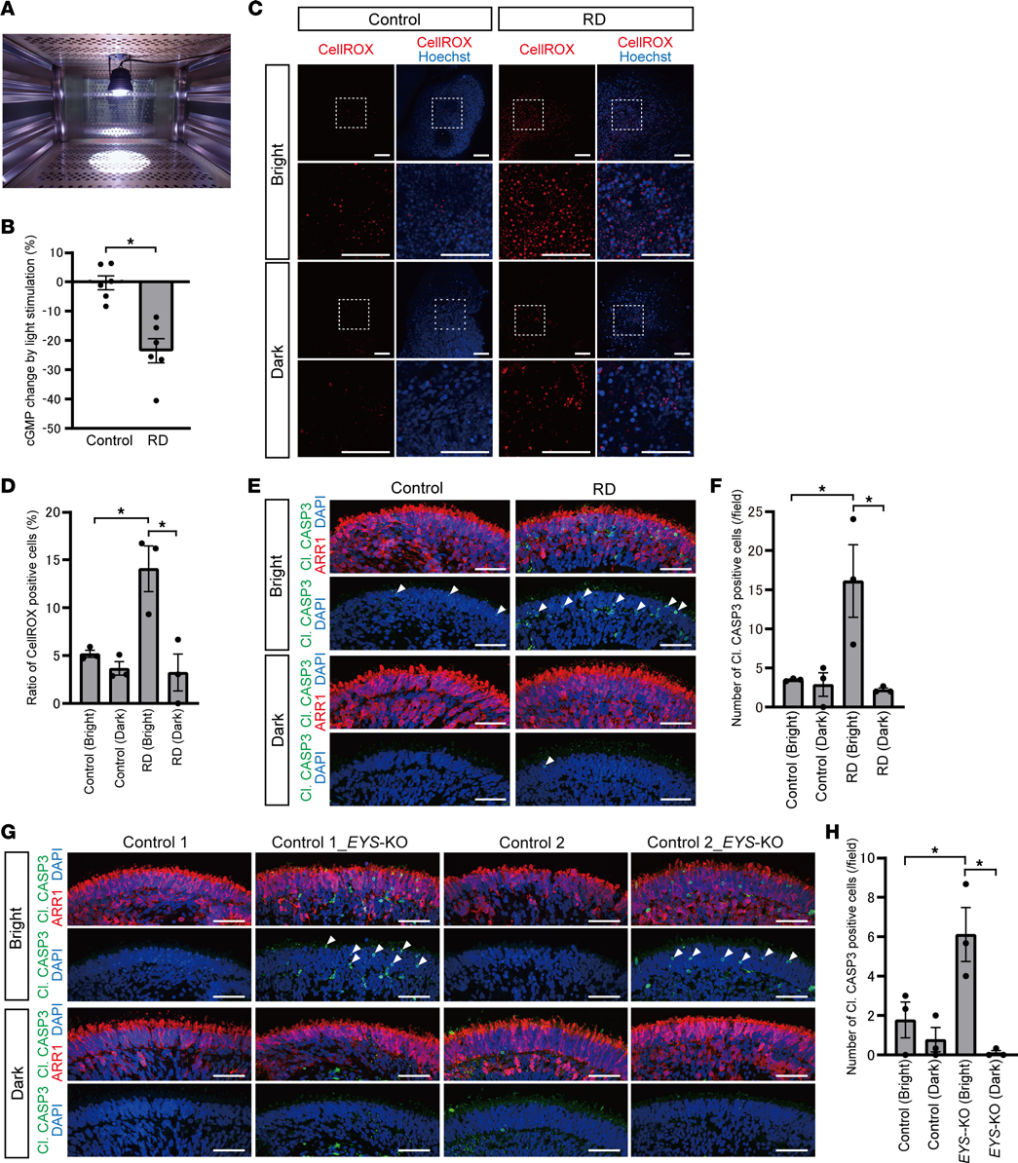
Light overresponse and photoreceptor cell death after light irradiation in RD retinal organoids. (**A**) Retinal organoids were exposed to white LED light on day 180. (**B**) Intracellular cGMP concentration was measured by ELISA. Percentage changes in cGMP concentration by light stimulation in control and RD retinal organoids are shown. Data represent mean ± SEM from independent experiments (*n* = 6). An unpaired, 2-tailed *t* test was used for statistical comparison (*P < 0.05). (**C**) Representative images of reactive oxygen species (ROS) stained with CellROX in control and RD retinal organoids. Twenty-four-hour light-stimulated (Bright) or dark-adapted (Dark) organoids on day 180 were evaluated. The lower panels are higher-magnification images of the dotted boxes in the upper panels. Scale bars: 100 μm. (**D**) Quantification of the data in **C**. The *y* axis indicates the ratio of CellROX-positive cells. Data represent mean ± SD from 3 retinal organoids. One-way ANOVA with Dunnett’s post hoc test was used for statistical comparison (*P < 0.05). (**E**) Representative immunofluorescence images of photoreceptor marker ARR1 and cleaved caspase-3 (Cl. CASP3) in retinal organoids after light exposure or dark adaptation. White arrowheads indicate cleaved caspase-3–positive cells. Scale bars: 50 μm. (**F**) Quantification of the data in **E**. The *y* axis indicates the number of cleaved caspase-3–positive cells per field. Data represent mean ± SEM from 3 retinal organoids. One-way ANOVA with Dunnett’s post hoc test was used for statistical comparison (*P < 0.05). (**G**) Representative immunofluorescence images of ARR1 and cleaved caspase-3 in control and *EYS*-KO retinal organoids after light exposure or dark adaptation. White arrowheads indicate cleaved caspase-3–positive cells. Scale bars: 50 μm. (**H**) Quantification of the data in **G**. The *y* axis indicates the number of cleaved caspase-3–positive cells per field. Data represent mean ± SEM from 3 retinal organoids. One-way ANOVA with Dunnett’s post hoc test was used for statistical comparison (*P < 0.05).

GRKs phosphorylate and deactivate opsins in the OS, contributing to the shutoff of the photoresponse (Supplemental Figure 7A). This prompted us to study whether GRK deprivation in the OS in RD organoids would lead to a different light response. We evaluated this phenomenon by measuring the cyclic GMP (cGMP) level by enzyme-linked immunosorbent assay (ELISA) analysis in control and RD organoids, depending on the light-stimulated or dark-adapted conditions. On day 179, the medium was changed to one without phenol red to avoid light absorption, and the vitamin A analog 9-*cis*-retinal (10 μM) was added to activate a phototransduction cascade (38). On the following day, organoids were exposed to white LED light for 1 hour before evaluation (Figure 6A). As a control, overnight dark–adapted organoids were also collected under dim red light. The intracellular cGMP level in RD was reduced by light irradiation, whereas that in control hardly changed between light-stimulated or dark-adapted conditions. The decrease in cGMP induced by light exposure was significantly greater in RD than in control (Figure 6B). We confirmed that 1-hour light stimulation did not cause obvious cell death in RD organoids by immunostaining for cleaved caspase-3 (Supplemental Figure 7B). We also tested the cGMP level using retinal organoids without 9-*cis*-retinal treatment as a control. We found that groups without 9-*cis*-retinal treatment did not exhibit any obvious change in cGMP concentration after light stimulation (Supplemental Figure 7C).

Next, to test whether light overresponse due to GRK7 deprivation from the OS can cause photoreceptor cell death, we examined light-induced damage with retinal organoids. Light exposure and dark adaptation processes were conducted as well as ELISA, but exposure duration was extended to 24 hours. First, we measured reactive oxygen species (ROS) using CellROX reagent and compared its level among light-stimulated or dark-adapted control and RD organoids (Figure 6C). The ratio of CellROX-positive cells was increased by light exposure in RD organoids, and it was significantly higher in light-exposed RD organoids than in any other groups (Figure 6D).

Second, we evaluated photoreceptor cell death induced by light exposure with staining for cleaved caspase-3. Cleaved caspase-3–positive cells in the photoreceptor cell layer were barely detected in dark-adapted control and RD organoids (Figure 6E). On the other hand, they appeared much more frequently in RD after light exposure than in control (Figure 6E). The quantified data revealed a similar trend to that of CellROX analysis in that the number of cleaved caspase-3–positive cells was most frequently detected in light-exposed RD organoids, and there were significant differences from the other groups (Figure 6F). We confirmed that no cleaved caspase-3–positive cells were detected in a layer in which photoreceptor cells were absent (Supplemental Figure 7D). We tested whether photoreceptor cell death induced by light exposure was also observed in *EYS*-KO organoids. Cleaved caspase-3–positive cells in the photoreceptor cell layer were frequently detected in light-exposed *EYS*-KO organoids as well as in RD organoids (Figure 6G). The quantified data showed that there were significant differences from the other groups (Figure 6H). Collectively, we confirmed that the phenotypes of GRK7 mislocalization and light-induced cell death also occurred in *EYS*-KO photoreceptor cells.

### Light-induced photoreceptor cell death in RD organoids is especially caused by blue light.

**Figure 7:**
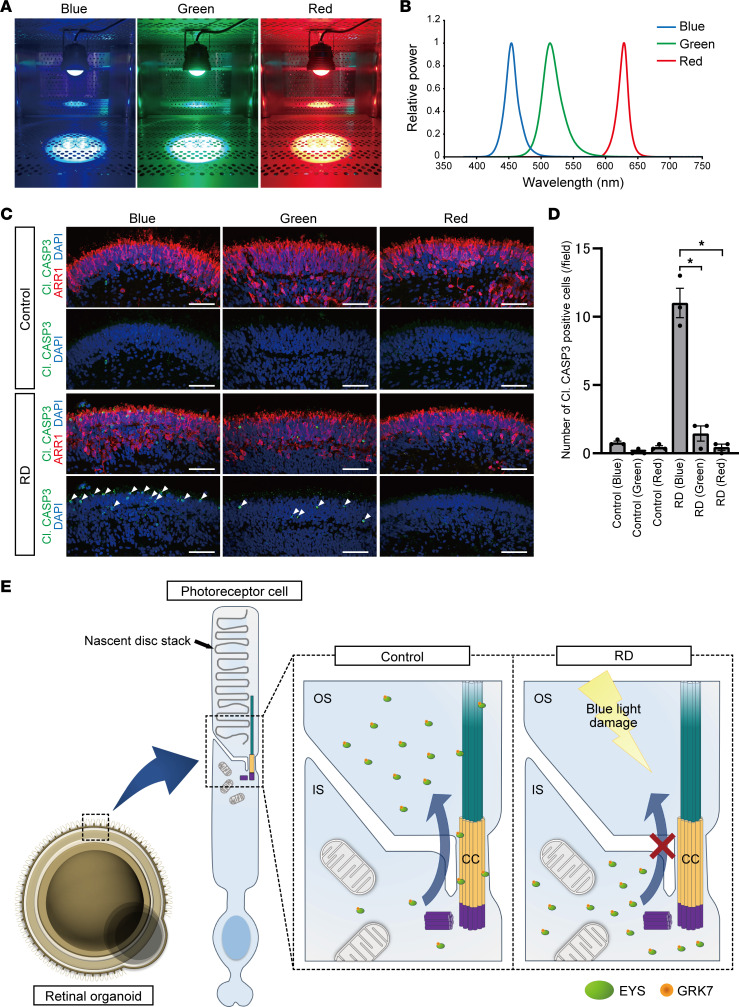
The effect of light wavelength on photoreceptor cell death in retinal organoids. (**A**) Retinal organoids were exposed to blue, green, or red LED light on day 180 for 24 hours. (**B**) The peak wavelengths of blue, green, and red lights were 454, 514, and 628 nm, respectively. (**C**) Representative immunofluorescence images of photoreceptor marker (ARR1) and cleaved caspase-3 (Cl. CASP3) in control and RD retinal organoids after exposure to each LED light. White arrowheads indicate cleaved caspase-3–positive cells. Scale bars: 50 μm. (**D**) Quantification of the data in **C**. The *y* axis indicates the number of cleaved caspase-3–positive cells per field. Data represent mean ± SEM from 3 retinal organoids. One-way ANOVA with Dunnett’s post hoc test was used for statistical comparison (*P < 0.05). (**E**) Schematic representation of putative mechanism of *EYS*-RD. EYS interacts with GRK7 and transports it from the IS to the OS. In control photoreceptor cells, EYS can localize at the CC and OS, whereas in RD, EYS with GRK7 cannot distribute to these regions. Light-induced damage via excessive reactive oxygen species production is caused by the mislocalization of mutant EYS with GRK7.

To identify the wavelength that largely affects photoreceptor cell death, retinal organoids on day 180 were exposed to blue, green, or red light for 24 hours (Figure 7A). The peak wavelengths of the respective colors were 454 nm, 514 nm, and 628 nm (Figure 7B), and the illuminance of each light was adjusted to 1,000 lux. We found that cleaved caspase-3–positive cells were frequently detected in the photoreceptor layer of blue light–exposed RD organoids (Figure 7C). Quantitative analysis showed that the number of cleaved caspase-3–positive cells was significantly greater in blue light–exposed RD organoids than in other groups (Figure 7D). In addition, retinal organoids were also irradiated by the 3 colors at the same power (15 mW/cm^2^). Although cleaved caspase-3–positive cells tended to be detected most frequently after blue light exposure, there was no significant difference between blue and green light (Supplemental Figure 7, E–H). We summarized the putative pathogenic mechanisms of *EYS*-RD in Figure 7E.

## Discussion

Here, we established a 3-dimensional RD model by using iPSCs derived from patients with *EYS*-RD. We found that mutant EYS mislocalized in the cytoplasm of the IS or outer nuclear layer (ONL) in RD photoreceptor cells, whereas healthy control EYS localized at the CC and OS. EYS bound to one of the OS proteins, GRK7, and transported it to the OS. In RD organoids, GRK7 dysfunction caused by mutant EYS led to the overresponse to light stimuli and resulted in cell death via ROS generation. GRK7 mislocalization with light-induced photoreceptor cell death was also observed in *EYS*-KO organoids and *eys*-KO zebrafish. Furthermore, light-induced cell death in RD organoids was especially caused by short-wavelength light. These data revealed physiological EYS effects on the photoresponse in human photoreceptor cells and pathogenic mechanism in *EYS*-RD.

To reveal the molecular mechanism underlying *EYS*-RD, we generated *EYS*-KO iPSCs and confirmed that common phenotypes detected in RD organoids were also observed in *EYS*-KO organoids. Moreover, the phenotypes of EYS and GRK7 mislocalization were rescued by delivering control EYS to RD organoids. Although adeno-associated virus (AAV) vectors are the most widely used in both basic research and clinical settings, they have a relatively small packaging capacity of 5 kb. Because of the large size of the *EYS* gene, we employed HDAdV, which does not contain all viral genes and has a packaging capacity of 36 kb, providing long-term transgene expression (39). We also found that a gain-of-toxic-function mechanism by mutant EYS was not detected in EYS-overexpression experiments. These results demonstrated that RD pathogenesis is caused by mutant EYS mislocalization via a loss-of-function mechanism.

The localization of EYS protein has been investigated in the retina of zebrafish, monkeys, and humans. All studies consistently showed that EYS was localized at the basal end of acetylated α-tubulin, corresponding to the CC (10, 34, 35). We also confirmed EYS localization in the retina of human organoids, zebrafish, and a pig. CC links the IS and OS in photoreceptor cells, and all OS components are transported via the CC from the site of synthesis in the IS (40). Considering its localization, EYS is expected to be related to OS protein trafficking. Moreover, EYS has also been detected at the ciliary axoneme and OS in monkeys and humans (10, 14). In human retinal organoids, EYS dominantly existed around the cilia and nascent OS regions, recapitulating human retina of previous reports. Unlike mammals, teleost fish, including zebrafish, harbor an accessory OS, which is believed to correspond to the mammalian ciliary axoneme but is separated from the OS except for thin plasma bridges enclosed by a continuous membrane (41). The different EYS staining pattern between zebrafish and mammals can be explained by the anatomical differences in photoreceptor cells. Collectively, EYS dominantly exists from cilia to OS regions, indicating that EYS plays a role in transporting some specific proteins to the OS.

We successfully detected both intracellular full-length and truncated mutant EYS by Western blot analysis and found that human EYS dominantly existed at the cilia and OS, whereas mutant EYS was less frequently observed in these regions. This mislocalization is consistent with a previous report describing *eys-*mutant zebrafish with c.5577_5584del in exon 34 (11). It demonstrated that mutant Eys was not detected around the CC but dominantly existed in the ONL or synaptic terminals. Regarding the molecular mechanism of EYS localization to the CC, it requires binding with matriglycans via its laminin G domain near the C-terminus (34). Therefore, mutant EYS was not capable of reaching the CC and OS due to its lack of a C-terminus, and this might lead to losing its ability to regulate specific OS protein trafficking.

Previous studies reported the mislocalization of several OS proteins such as rhodopsin (12, 35) and cone opsin (11) in *eys*-KO zebrafish. However, direct interaction between Eys and such proteins has not been reported to the best of our knowledge, and quantitative analysis was absent except for manual counting. Therefore, we examined direct interactions with human EYS protein and its localization in organoids by an unbiased method. In previous reports using retinal organoids, OS protein mislocalization was evaluated by a ratio comparing the fluorescence intensity between the IS plus OS regions and the ONL region (25). This method is suitable for estimating post-Golgi transport, but is insufficient for cilia trafficking, as IS and OS regions were not discriminated. In the present study, we selected PRPH2 to show the OS region of photoreceptor cells in organoids. This is because the photoreceptor cilium lacking PRPH2 fails to elaborate an OS (42), but *eys*-KO zebrafish exhibit morphologically normal disc stacks in their early stage (11). We confirmed that PRPH2 was transported to the OS independently of EYS by IP and immunocytochemical analysis. In this way, we successfully identified the OS and evaluated the target protein localization by performing double staining for PRPH2.

GRKs phosphorylate activated G protein–coupled receptors (GPCRs) and regulate their sensitivity (43). Although there are 7 mammalian GRKs, *GRK1* and *GRK7* are expressed specifically in photoreceptor cells. *GRK7* is virtually specific to cone cells, whereas *GRK1* is expressed in both rods and cone cells (44). Human *EYS* is expressed more dominantly in cone cells than rod cells, as is *GRK7* (Human Protein Atlas; https://www.proteinatlas.org/ENSG00000188107-EYS/single+cell+type), and *eys*-KO zebrafish exhibit cone-dominant photoreceptor loss (11). Although human *EYS* variants usually cause RP, in some patients cone cell degeneration precedes that of rod cells, exhibiting cone rod dystrophy (45) or macular dystrophy (46). With respect to evolutionary biology, the *EYS* gene is dispensed in 4 independent mammalian lineages, including some rodents (mouse, rat, guinea pig, and rabbit), armadillos (*Dasypus novemcinctus*), little brown bats (*Myotis lucifugus*), and ruminants (cattle and sheep) (2), whereas *EYS* is conserved in some rodents like ground squirrels. This suggests that the *EYS* gene tends to be essentially lost in nocturnal animals. Notably, both *EYS* and *GRK7* are lost in some common mammals such as rodents (mouse, rat), armadillos, and bats (*Myotis davidii*, *Myotis lucifugus*) (47–49). This common trend should be remarkable, because their rod-dominant retinas even spare some cone cells. Based on this evidence indicating that EYS is related to daylight vision (cone cell function), we focused on GRK7 as a counterpart of the EYS protein. We found that EYS interacts with GRK7, and GRK7 is dislocated from the OS in RD organoids and *eys*-KO zebrafish, although the binding capacity for GRK7 was not different between healthy control and mutant EYS. These results indicate that EYS and GRK7 may have evolved together in response to light in the evolutionary process, and the pathogenic mechanism underlying RD is that the EYS-GRK7 complex is not localized in the OS via the CC and cannot respond to light properly.

In the photoreceptor OS, GRK1 and/or GRK7 phosphorylate and deactivate light-activated opsins, contributing to light adaptation (47). Unlike GRK1, the amount of research on GRK7 is still limited because of its lack of rodent models, as is true of EYS. Although there are several reports describing *grk7*-knockdown (50, 51) or -KO (52) zebrafish, all of them focused on their impaired response recovery without long-term observation and did not investigate light-induced damage or retinal degeneration. In general, cone cells are not saturated as easily as rod cells and continue to respond even under bright illumination (53). Therefore, deprivation of GRK7 in the OS may lead to higher and longer transducin activation and finally intense light-induced damage (28). We measured intracellular cGMP levels to evaluate the changes under light conditions. cGMP is one of the essential components in the phototransduction cascade, and light stimulation leads to a decrease in its concentration by activating phosphodiesterase molecules (54). In the current study, RD organoids exhibited a significant decrease in cGMP concentration compared with controls after light exposure. This indicates that GRK7 dysfunction in RD photoreceptor cells strongly induced more activation of the downstream cascade, resulting in massive cGMP degradation.

The effect of light on IRD patients is still controversial because controlling light exposure is not easy, particularly when a long-term study is needed (55). In addition, response to light exposure may differ, depending on the causative genes. In any event, it is challenging to analyze a certain number of patients carrying causative variants in a specific gene. In some animal models, however, an inferior-dominant sector pattern of photoreceptor loss was observed, and light deprivation reduces retinal degeneration (56, 57). In fact, it was reported that patients with pathogenic variants in some specific genes, including *EYS* (58) and *RHO* (59), exhibit such patterns of retinal degeneration. Although the mechanism of photochemical damage has not been completely elucidated, the formation of ROS following light exposure is one of the major factors causing photoreceptor cell death (60). In the current study, photoreceptor cells in RD organoids exhibited a higher level of ROS production and cell death after light exposure than controls. These results indicate that the excessive activation of the phototransduction cascade caused much more ROS generation that overcame the protective mechanisms, thus resulting in cell death.

Due to technical limitations, however, we found that it was difficult to discriminate rod and cone photoreceptor cells and to identify the dying cell type in organoids. According to previous reports, white light exposure causes rod-dominant cell loss in albino and WT zebrafish (60–62). In contrast, the retina of *eys*-KO zebrafish was damaged even by shorter light exposure than that described in previous reports and exhibited cone-dominant cell death. These results are consistent with the degeneration pattern observed in *eys*-KO zebrafish (10, 11), and indicate that Eys played a crucial role in protecting cells from light-induced damage. Furthermore, transmission electron microscopy analysis revealed abnormalities in the OS structure in all light-exposed *eys*-KO zebrafish. Such disorganization of the normally neatly stacked OS was similar to the finding in light-irradiated murine retinal explant cultures described in a previous report (63). This suggested that the OS was the primary site of light damage that led to photoreceptor cell death. Collectively, these results demonstrated that cell death caused by light-induced cell damage is one of the major mechanisms in patients with *EYS*-RD.

Furthermore, we found that blue light largely causes photoreceptor cell death in RD retinal organoids. It has been reported that prolonged exposure to blue light can be harmful to photoreceptor cells in both in vitro (64, 65) and in vivo models (66, 67). Under the same illuminance, the energy of blue light is generally higher than that of other colors. Although the underlying mechanism of cell damage induced by blue light is not fully understood, short-wavelength light is believed to likely cause photochemical damage due to its higher energy (68). Considering that S-cones are the least numerous photoreceptors, accounting for several percentages of all cones (69) and retinal organoids, higher-energy light may affect other photoreceptor cell types. In the current study, blue light caused the greatest amount of photoreceptor cell death under the same illuminance, whereas cell damage did not differ under the same intensity. Therefore, these results indicate that intense-energy light highly drove the phototransduction cascade in light-sensitive RD organoids, which then led to excessive ROS production and photoreceptor cell death. Accordingly, since the illuminance of blue light tends to be underestimated by human eyes, cutting harmful short-wavelength light would be beneficial for patients with *EYS*-RD by using tinted glasses or other devices.

In summary, we show that an RD model with retinal organoids generated from patient-derived iPSCs is a useful tool for investigating the pathogenesis of *EYS*-RD. Our data demonstrate that photoresponse integrity is impaired by EYS mislocalization, and it would be a potential therapeutic approach for patients with *EYS*-RD to restrict the exposure to high-energy short-wavelength light.

### Limitations of the study.

First, our study is based on the analysis of a limited number of iPSC clones. Variability among iPSC clones might affect and/or cover the results to a certain extent. However, we could duplicate the phenotype that was found in RD retinal organoids by generation of *EYS*-KO retinal organoids and *eys*-KO zebrafish.

Second, the number of disc stacks in the OS is limited and they are not typically organized in retinal organoids (70). Although we confirmed the expression of an essential motor protein and a robust cilium structure by scanning electron microscopy, physiological conditions may not be completely recapitulated in retinal organoids. However, they provide useful information regarding EYS localization and functions, given that no mammalian models have been established, and photoreceptor cells are already disorganized in human retinas of most elderly cases.

Lastly, it is still unclear why *EYS* variants cause rod-dominant photoreceptor cell death, even though *EYS* and *GRK7* are more dominantly expressed in cone cells. The primary structure of Crumbs homolog 1 encoded by the *CRB1* gene is similar to EYS, in that they have repeats of multiple EGF-like and laminin A G–like domains (71), and both are involved in photoreceptor development according to a study in *Drosophila* (72). Although human EYS may play a role as a secreted extracellular matrix protein (34), both EYS and CRB1 are reported to localize at the CC in vertebrates. Such similarities indicate that they perform similar functions in photoreceptor cells. Despite its expression only in cone cells, Crb1 promoted rod survival under light irradiation in a non–cell-autonomous fashion (73). Such a mechanism may also affect rod cell survival and be related to rod-dominant degeneration in *EYS*-RD. Moreover, it is possible that EYS also performs functions especially in rod cells, including trafficking of other molecules, even though we could not detect a difference in staining pattern and the amount of other major photoresponse-associated proteins such as rhodopsin, RCVRN, GNAT2, and ARR1 in both organoid and zebrafish models.

## Methods

Further information can be found in Supplemental Methods. Detailed information for all antibodies used in this study is provided in Supplemental Table 2.

### Sex as a biological variable.

Sex was not considered as a biological variable because the *EYS* gene was identified as the autosomal recessive causative gene for RP (2).

### iPSCs and zebrafish maintenance.

The information on human iPSCs used in the present study is shown in Supplemental Table 1. Human iPSCs were maintained on laminin (iMatrix-511; TaKaRa) in StemFit AK02N medium (Ajinomoto) at 37°C in a standard 5% CO_2_ incubator, according to a published protocol (74). Passages were performed every 7 days. Six-well culture plates were coated with laminin in PBS at 37°C for at least 1 hour. iPSC colonies were treated with TrypLE Select Enzyme (Thermo Fisher Scientific) at 37°C for 4 minutes and dissociated into single cells by gentle pipetting. The dissociated iPSCs were suspended in StemFit medium with 10 μM Y-27632 (Rock inhibitor; Nacalai-Tesque) and counted with a Countess III Automated Cell Counter (Thermo Fisher Scientific). The single-cell iPSC suspension was plated at a density of 13,000 cells per well. On the following day, the medium was changed to StemFit without Y-27632 and then further changed every other day. Fish were raised under 12-hour light/12-hour dark (L/D) cycles during the first 5 days after birth, and after that at 14:10 L/D.

### Statistics.

We conducted the experiments with biological triplicates, and data are expressed as mean ± standard deviation (SD) or standard error of the mean (SEM). Results were analyzed using 1-way analysis of variance (ANOVA) followed by Dunnett’s post hoc test or unpaired *t* test to determine statistical significance of the data. All analyses were performed using SPSS Statistics 19 software (SPSS, Inc.), and statistical significance was defined as *P* less than 0.05.

### Study approval.

The use of iPSCs was approved by the Ethics Committees of the RIKEN BioResource Research Center, Kyoto University, and Kansai Medical University. All zebrafish experiments in the present study were carried out in accordance with the Institutional Animal Care and Use Committee (IACUC) of the National Institute of Genetics (NIG) of Japan (approval numbers R2-6, R3-1, and R4-7). All the experimental protocols were approved by the IACUC of the NIG. Pig experiments were approved by the IACUC of Meiji University (MUIACUC2020-125). All animal care and experimental procedures for pig experiments were performed in accordance with the Japan Act on Welfare and Management of Animals and all applicable regulations.

### Date availability.

Single-cell RNA sequencing data have been deposited in the NCBI Gene Expression Omnibus database (GEO GSE259339). All raw data that support the findings of this study are available in the supplemental Supporting Data Values file.

## Author contributions

YO, AO, KI, AT, and HI conceived the project. YO, RN, II, YS, K Tsukita, and NO performed experiments. K Teranaka, YN, and AW analyzed the single-cell RNA sequencing data. KU and HN prepared the pig retinas. KA and KK raised zebrafish and generated *eys*-KO zebrafish. YO and K Muguruma generated retinal organoids. K Mitani generated the adenoviral vector. YO, KI, AO, TK, MS, AT, and HI analyzed data and provided scientific discussions. YO, AO, KI, KA, K Mitani, and HI wrote the manuscript.

## Supplementary Material

Supplemental data

Supporting data values

## Version 1 04/22/2024

Electronic publication
