# Supplementary material for: Phototoxicity avoidance is a potential therapeutic approach for retinal dystrophy caused by EYS dysfunction
Source: JCI Insight. 2024 Apr 22;9(8):e174179. doi: 10.1172/jci.insight.174179 (PMC11141876; doi:10.1172/jci.insight.174179)
Supplement: Supplemental data [file jciinsight-9-174179-s055.pdf]

Supplemental Information

Title: Phototoxicity avoidance is a potential therapeutic approach for retinal dystrophy caused by EYS dysfunction

Authors:

Yuki Otsuka, Keiko Imamura, Akio Oishi, Kazuhide Asakawa, Takayuki Kondo, Risako Nakai, Mika Suga, Ikuyo Inoue, Yukako Sagara, Kayoko Tsukita, Kaori Teranaka, Yu Nishimura, Akira Watanabe, Kazuhiro Umeyama, Nanako Okushima, Kohnosuke Mitani, Hiroshi Nagashima, Koichi Kawakami, Keiko Muguruma, Akitaka Tsujikawa, Haruhisa Inoue

Supplemental Figure 1. Generation and characterization of iPSCs from patients with *EYS*-RD
Supplemental Figure 2. Single cell RNA sequencing with retinal organoids
Supplemental Figure 3. Differentiation status of retinal organoids on Day 180 was not different between Control and RD
Supplemental Figure 4. Anti-EYS antibody validation by EYS-overexpressed 293T cells
Supplemental Figure 5. Neither Peripherin-2 nor GRK1 interacted with EYS
Supplemental Figure 6. Additional results of *eys*-knockout zebrafish
Supplemental Figure 7. Additional results of light-induced damage on RD retinal organoids
Supplemental Figure 8. Full-length pictures of western blot analysis presented in the main and supplemental figures
Supplemental Figure 9. Full-length pictures of immunoprecipitation presented in the main and supplemental figures

Supplemental Table 1. List of human iPSCs
Supplemental Table 2. List of antibodies used for immunocytochemistry, immunohistochemistry and western blot (WB) analysis

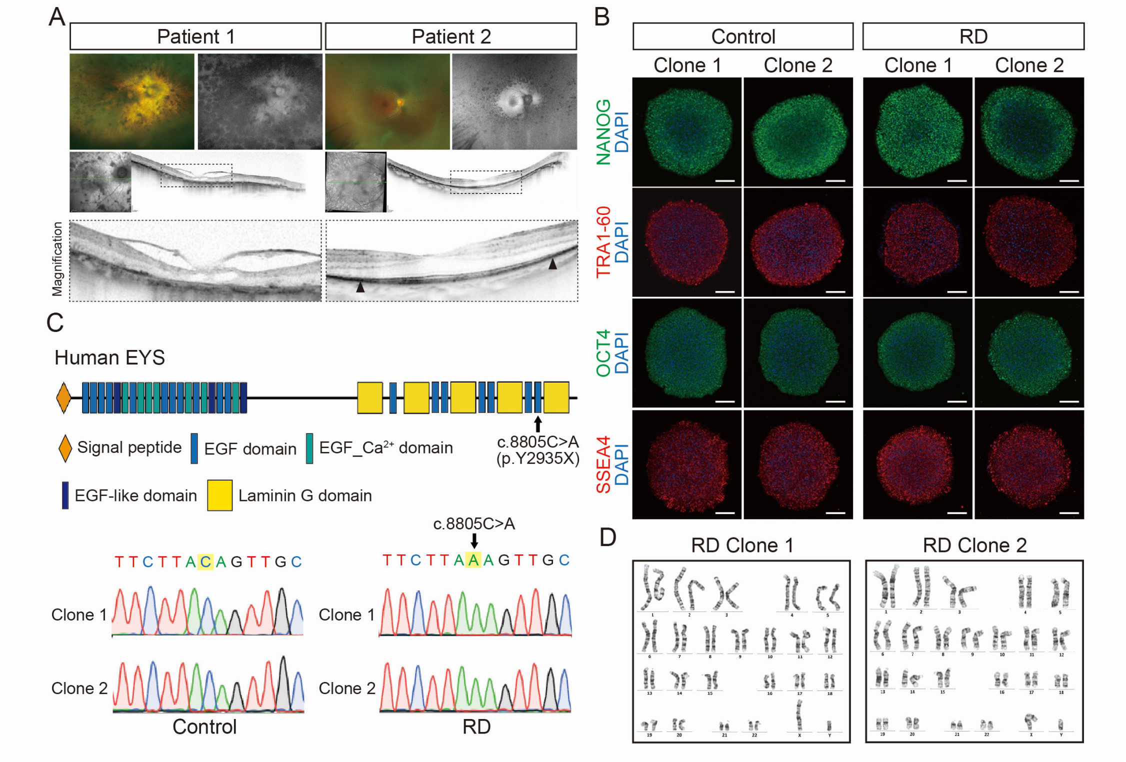

Figure S1. Generation and characterization of iPSCs from patients with *EYS*-RD, Related to Figure 1

(A) Fundus, autofluorescence and spectral-domain optical coherence tomography (SD-OCT) images of right eye in patients. Bottom panels show higher-magnification images of the dotted boxes in the upper SD-OCT panels. In RD patient 1, the ellipsoid zone (EZ) line was totally damaged in the macular region, whereas it was spared between the black arrowheads in RD patient 2.

(B) iPSCs from healthy individuals and patients with *EYS*-RD expressed pluripotent stem cell markers including NANOG, TRA1-60, OCT4 and SSEA4. Scale bars, 200 µm.

(C) A schematic image showing domain structures in human full length EYS protein. The mutation of c.8805C>A (p.Y2935X) is located in the last Epidermal Growth Factor (EGF) domain. Sanger sequencing shows that RD iPSC clones carried the homozygous mutations. EGF_Ca^{2+} domain: Calcium binding EGF domain.

(D) Both iPSC clones established from patients showed normal karyotypes.

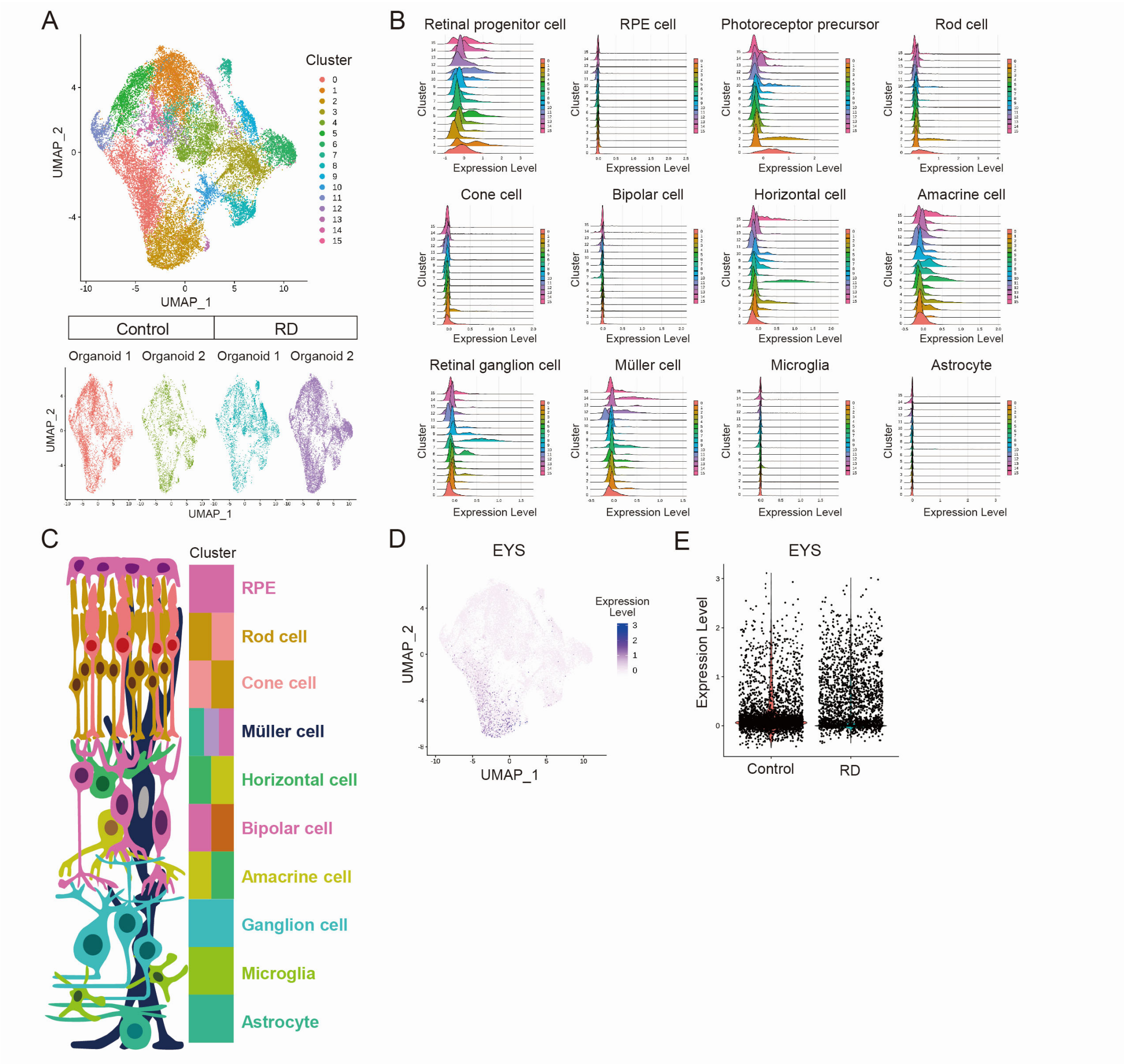

Figure S2. Single cell RNA sequencing with retinal organoids, Related to Figure 1

(A) Dimension reduction and clustering (PCA-UMAP) in all cells of retinal organoids on Day 70. Each point represents the transcriptome of a single cell. Component cell distributions on each clone are shown in the lower panel. Control organoid 1, HPS0063; Control organoid 2, HPS1042; RD organoid 1, HPS3927; RD organoid 2, HPS3933.

(B) Major retinal component cells are displayed in each panel. The expression levels of their representative markers described below are shown in each cluster. Retinal progenitor cells: LHX2, PAX6 and VSX2; RPE cells: BEST1, MITF, RPE65 and TRPM1; Photoreceptor precursors: CRX, PRDM1, OTX2 and RCVRN; Rod cells: CNGA1, CNGB1, NR2E3, NRL, RHO and SAG; Cone

cells: ARR3, CNGA3, CNGB3, GNAT2, OPN1MW and OPN1SW; Bipolar cells: GRIK1 and PKCA, PCP2 and TRPM1; Horizontal cells: CALB2, LHX1, ONECUT1, ONECUT3 and TFAP2B; Amacrine cells: FILIP1L, GAD1, GAD2, ISL1, MEIS2 and TFAP2A; Retinal ganglion cells: NEFL, POU4F1, POU4F2, RBPMS and SNCG; Müller glia: APOE, COL9A1, ITM2B, RLBP1, S100A16 and SLC1A3; Microglia: AIF1 and ICAM1; Astrocytes: GFAP.

(C) A schematic image of retinal component cells in the human retina. Each cell is colored by its most dominant cluster corresponding to (A) and (B).

(D) EYS-expressed cells are shown in UMAP. Each point represents the transcriptome of a single cell.

(E) EYS expression level in each cell was shown in Control and RD. The vertical axis indicates the expression level. Each point represents a single cell.

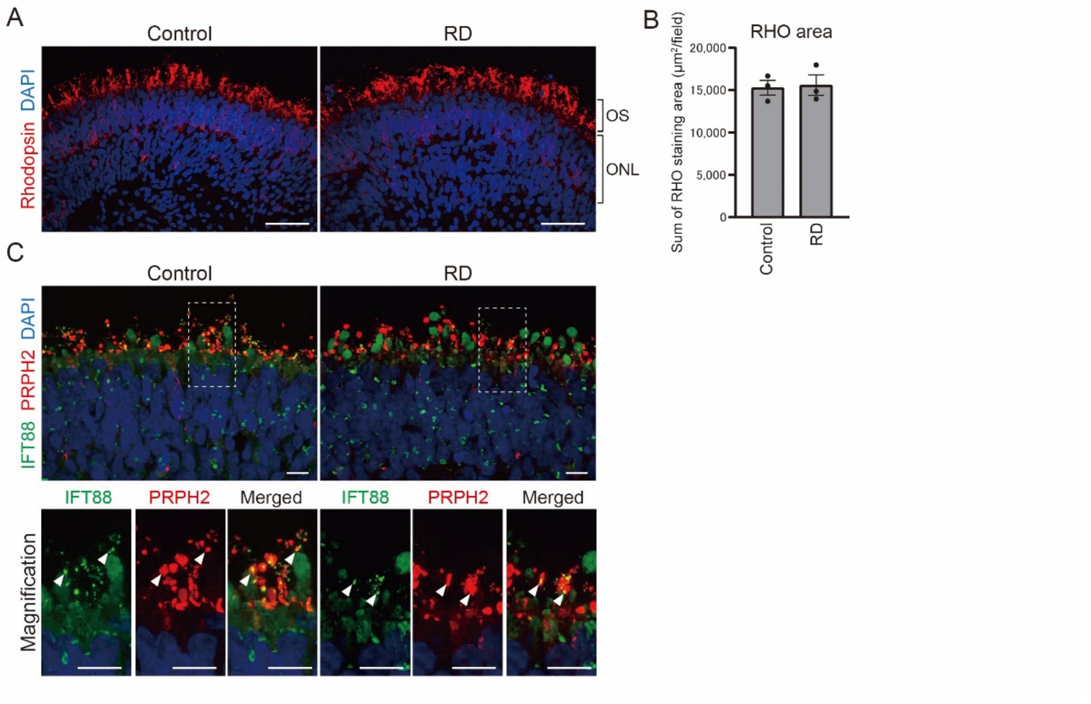

Figure S3. Differentiation status of retinal organoids on Day 180 was not different between Control and RD, Related to Figure 1

(A) Representative immunofluorescence images of Control and RD retinal organoids with Rhodopsin (RHO) on Day 180. ONL: outer nuclear layer; OS: outer segment. Scale bars, 50 µm.

(B) Quantitative data of RHO staining dimension in the surface of retinal organoids. The vertical axis indicates the sum of RHO staining area (µm^2) per field. Data represent mean ± SEM (n=3 organoids). Unpaired t-test was used for statistical comparison (p=0.85).

(C) Representative immunofluorescence images of retinal organoids with intraflagellar transport protein 88 (IFT88) and OS marker (Peripherin-2; PRPH2) on Day 180. Lower panels are higher-magnification images of the dotted boxes in the upper panels. White arrowheads indicate the connecting cilia located proximally at the OS. Scale bars, 10 µm.

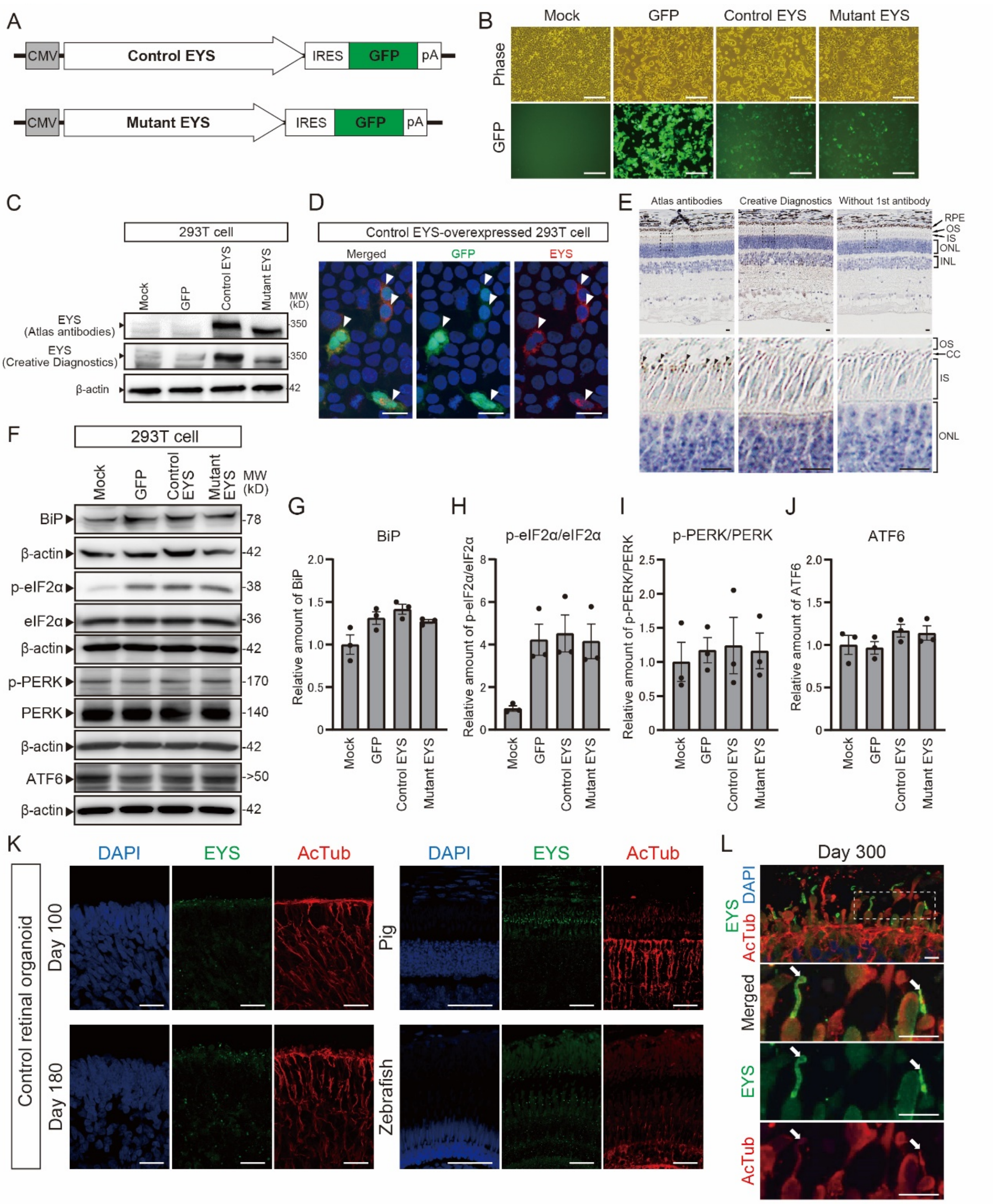

Figure S4. Anti-EYS antibody validation by EYS-overexpressed 293T cells, Related to Figure 2

(A) Design of a vector for Control or Mutant EYS overexpression. Control EYS represents full-length EYS cDNA, and Mutant EYS is a truncated one at c.8805. CMV: cytomegalovirus immediate early promoter; IRES: Internal Ribosome Entry Sites.

(B) Bright field view and GFP fluorescence of 293T cells 36 hours after transfection with vehicle, GFP, Control or Mutant EYS with IRES sequence and GFP. Scale bars, 200 μm.

(C) Western blot analysis using transfected 293T cells with commercially available anti-EYS antibodies from Atlas Antibodies and Creative Diagnostics. Approximately 325 kilodalton (kD) mutant EYS was also recognized and detected. Full-length picture of the blot is presented in Supplemental Figures 8M-O.

(D) GFP fluorescence and EYS immunostaining of 293T cells overexpressed by Control EYS with GFP. White arrowheads show GFP-positive cells with EYS staining. Scale bars, 20 μm.

(E) Representative images of immunohistochemistry (diaminobenzidine (DAB) staining), using wild type pig retina with anti-EYS antibodies (Atlas antibodies and Creative Diagnostics). Black arrowheads show EYS staining around connecting cilia (CC). RPE: retinal pigment epithelium; IS: inner segment; OS: outer segment; ONL: outer nuclear layer; INL: inner nuclear layer. Scale bars, 10 μm.

(F) Western blot analysis using transfected 293T cells with anti-Binding immunoglobulin protein (BiP), Eukaryotic initiation factor 2α (eIF2α), phospho-eIF2α (p-eIF2α), PKR-like endoplasmic reticulum kinase (PERK), phospho-PERK (p-PERK) and activating transcription factor 6 (ATF6). Full-length picture of the blot is presented in Supplemental Figures 8P-Y.

(G) Quantification of western blot bands in (F). The vertical axis indicates the relative amount of BiP. Data represent mean ± SEM from independent experiments (n=3). One-way ANOVA was used for statistical comparison.

(H) Quantification of western blot bands in (F). The vertical axis indicates the relative amount of p-eIF2α/eIF2α. Data represent mean ± SEM from independent experiments (n=3). One-way ANOVA was used for statistical comparison.

(I) Quantification of western blot bands in (F). The vertical axis indicates the relative amount of p-PERK/PERK. Data represent mean ± SEM from independent experiments (n=3). One-way ANOVA was used for statistical comparison.

(J) Quantification of western blot bands in (F). The vertical axis indicates the relative amount of ATF6. Data represent mean ± SEM from independent experiments (n=3). One-way ANOVA was used for statistical comparison.

(K) Separated channel immunofluorescence images of Control retinal organoids on Day 100 and Day 180, wild-type pig retina and wild-type zebrafish retina with DAPI, EYS and Acetylated α-Tubulin (AcTub), shown in Figure 2. Scale bars, 20 µm.
(L) Representative immunofluorescence images of EYS and AcTub in Control retinal organoids on Day 300. Lower panels are higher-magnification images of the dotted box in the upper panel. White arrows indicate the outer segment. Scale bars, 10 µm.

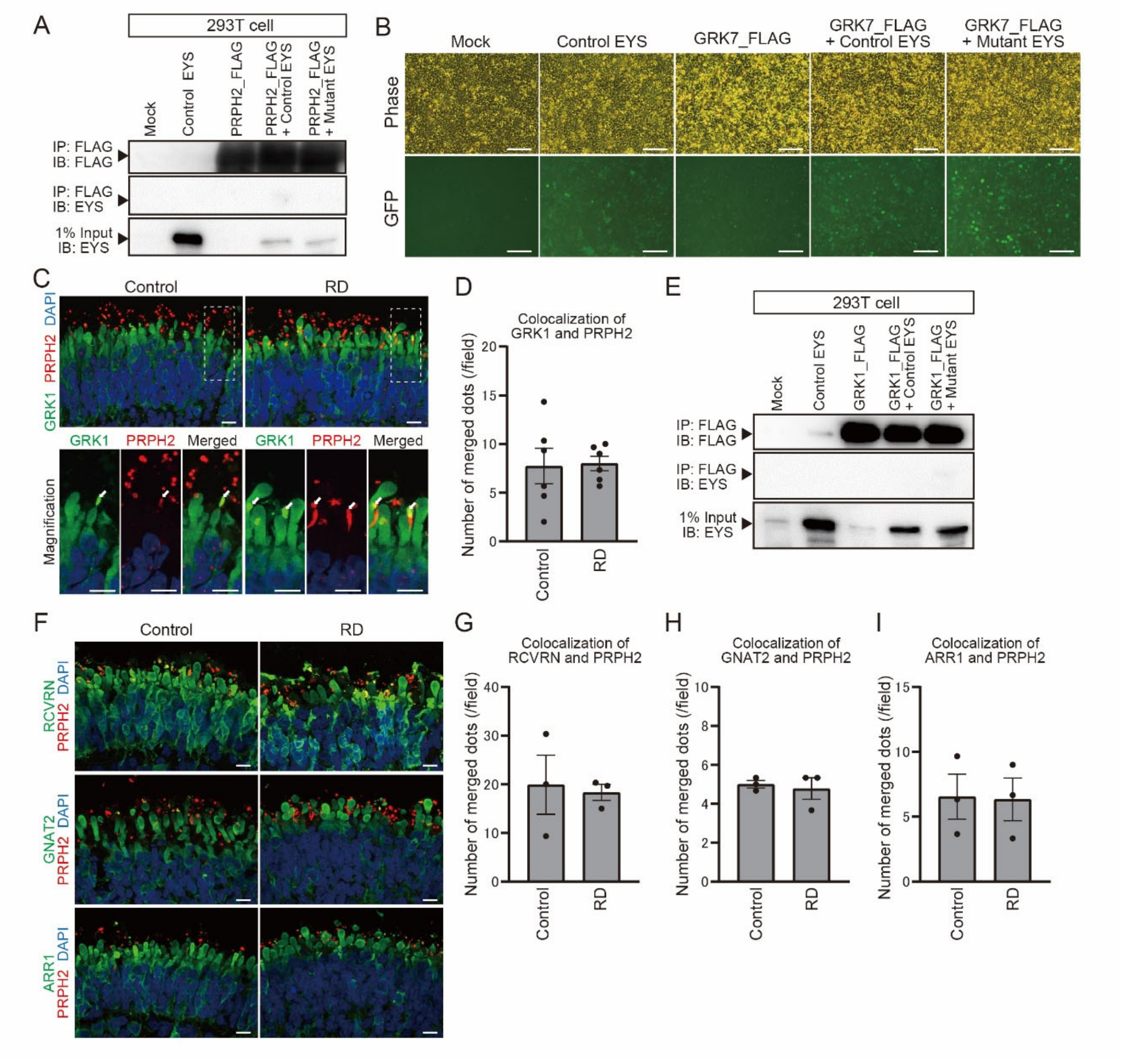

Figure S5. Neither Peripherin-2 nor GRK1 interacted with EYS, Related to Figure 3

(A) Immunoprecipitation (IP) of EYS and Peripherin-2 (PRPH2). Anti-FLAG antibody was used for IP, and immunoblotting (IB) was performed with antibodies against FLAG (top) or EYS (middle and bottom). Full-length picture of the blot is presented in Supplemental Figure 9B.

(B) Bright field view and GFP fluorescence of 293T cells 36 hours after lipofection. They were transfected with vehicle, Control EYS with Internal Ribosome Entry Sites (IRES) and GFP (Control EYS-GFP), GRK7 with FLAG (GRK7_FLAG), both Control EYS-GFP and GRK7_FLAG, and both Mutant EYS (Mutant EYS-GFP) and GRK7_FLAG. Scale bars, 200 µm.

(C) Representative immunofluorescence images of Control and RD retinal organoids on Day 180 with GRK1 and PRPH2. Lower panels are higher-magnification images of the dotted boxes in the upper panels. White arrows indicate OS. Scale bars, 10 μm.
(D) Quantitative analysis of merged immunoreactivity of PRPH2 and GRK1 in Control and RD retinal organoids. The vertical axis indicates the number of merged dots per field. Data represent mean ± SEM (n=6 organoids). Unpaired t-test was used for statistical comparison (p=0.92).
(E) IP of EYS and GRK1. Anti-FLAG antibody was used for IP, and IB was performed with antibodies against FLAG (top) or EYS (middle and bottom). Full-length picture of the blot is presented in Supplemental Figure 9C.
(F) Representative immunofluorescence images of Recoverin (RCVRN), G Protein Subunit Alpha Transducin 2 (GNAT2) and Arrestin1 (ARR1) with OS marker, PRPH2 in Control and RD retinal organoids on Day 180. Scale bars, 10 μm.
(G) A quantitative analysis of merged immunoreactivity of RCVRN and PRPH2 in Control and RD retinal organoids in (F). The vertical axis indicates the number of reactivity dots per field. Data represent mean ± SEM (n=3 organoids). Unpaired t-test was used for statistical comparison.
(H) A quantitative analysis of merged immunoreactivity of GNAT2 and PRPH2 in Control and RD retinal organoids in (F). The vertical axis indicates the number of reactivity dots per field. Data represent mean ± SEM (n=3 organoids). Unpaired t-test was used for statistical comparison.
(I) A quantitative analysis of merged immunoreactivity of ARR1 and PRPH2 in Control and RD retinal organoids in (F). The vertical axis indicates the number of reactivity dots per field. Data represent mean ± SEM (n=3 organoids). Unpaired t-test was used for statistical comparison.

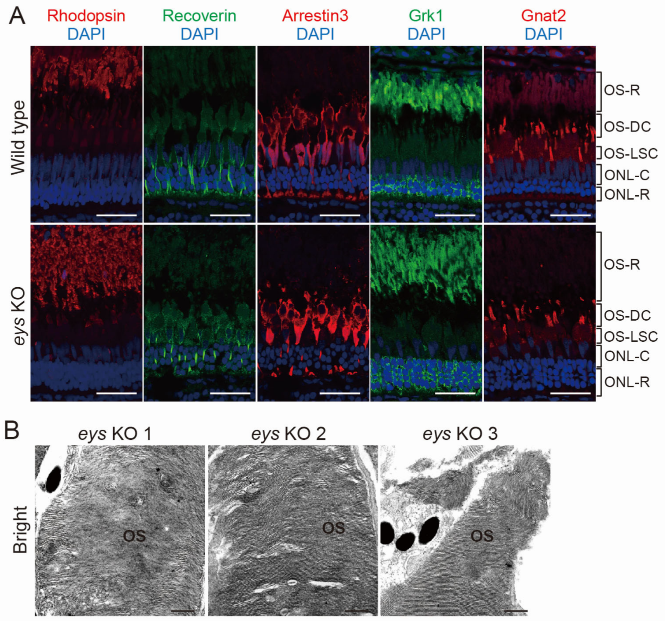

Figure S6. Additional results of *eys*-knockout zebrafish, Related to Figures 5

(A) Representative immunofluorescence images of wild-type and *eys*-KO zebrafish with Rhodopsin, Recoverin, Arrestin3, Grk1 and Gnat2. Scale bars, 20 μm.

(B) Representative transmission electron microscopic images of disorganized photoreceptor outer segment (OS) in three different *eys*-KO zebrafish. Scale bars, 500 nm.

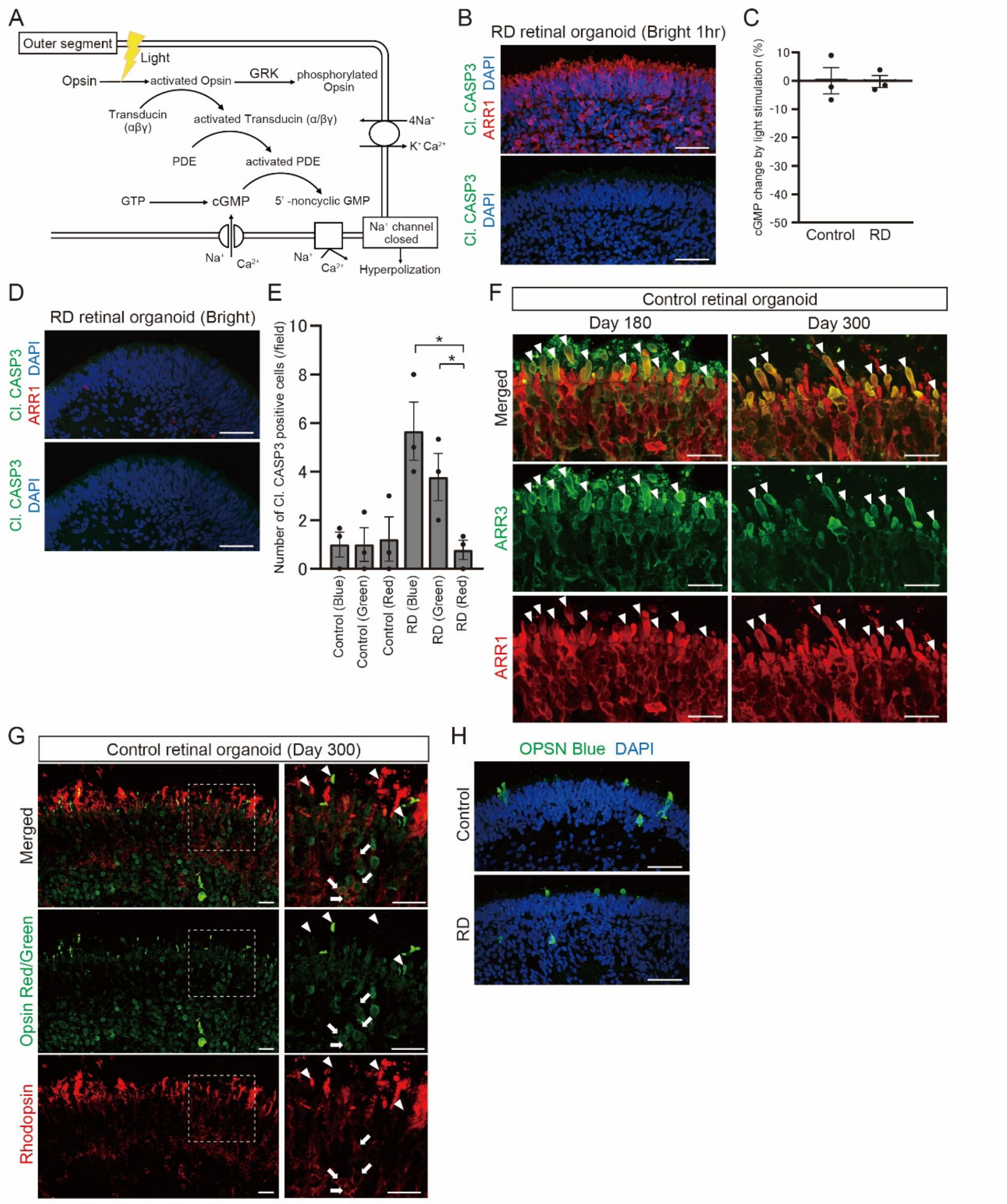

Figure S7. Additional results of light-induced damage on RD retinal organoids, Related to Figures 6 and 7

(A) A schematic image of phototransduction cascade in photoreceptor outer segment. GRK: G-protein-coupled receptor kinase; cGMP: cyclic GMP; PDE: Phosphodiesterase

(B) Representative immunofluorescence images of one hour light-irradiated RD retinal organoids on Day 180 with photoreceptor marker (ARR1) and cleaved caspase-3 (Cl. CASP3). Scale bars, 50 μm.

(C) Intracellular cGMP concentration was measured by Enzyme-linked immunosorbent Assay (ELISA). 9-cis Retinal was not added to all retinal organoids before the experiment. Percentage changes of cGMP concentration by light stimulation in Control and RD retinal organoids are shown. Data represent mean ± SEM from independent experiments (n=3). Unpaired t-test was used for statistical comparison.

(D) Representative immunofluorescence images of light-irradiated RD retinal organoids on Day 180 with photoreceptor marker (ARR1) and Cl. CASP3. Neural retinal layer is not well formed in the panels. Scale bars, 50 μm.

(E) Quantitative data of immunofluorescence images of ARR1 and Cl. CASP3 in Control and RD retinal organoids after 15 mW/cm^2 blue, green or red light exposure. The vertical axis indicates the number of cleaved caspase-3 positive cells per field. Data represent mean ± SEM from three retinal organoids. One-way ANOVA was used for statistical comparison (*$p<0.05$).

(F) Representative immunofluorescence images of Control retinal organoids on Day 180 and 300 with rod cell marker (Arrestin1; ARR1) and cone cell marker (ARR3). White arrowheads show photoreceptor cells stained with both ARR1 and ARR3. Scale bars, 20 μm.

(G) Representative immunofluorescence images of Control retinal organoids on Day 300 with rod cell marker (Rhodopsin) and cone cell marker (Opsin Red/Green). The right panels are higher-magnification images of the dotted boxes in the left panels. White arrowheads show the outer segment (OS) of photoreceptor cells stained by either Rhodopsin or Opsin Red/Green. White arrows show cytoplasm stained by both Rhodopsin and Opsin Red/Green. Scale bars, 20 μm.

(H) Representative immunofluorescence images of Control and RD retinal organoids on Day 180 with Opsin Blue. Scale bars, 50 μm.

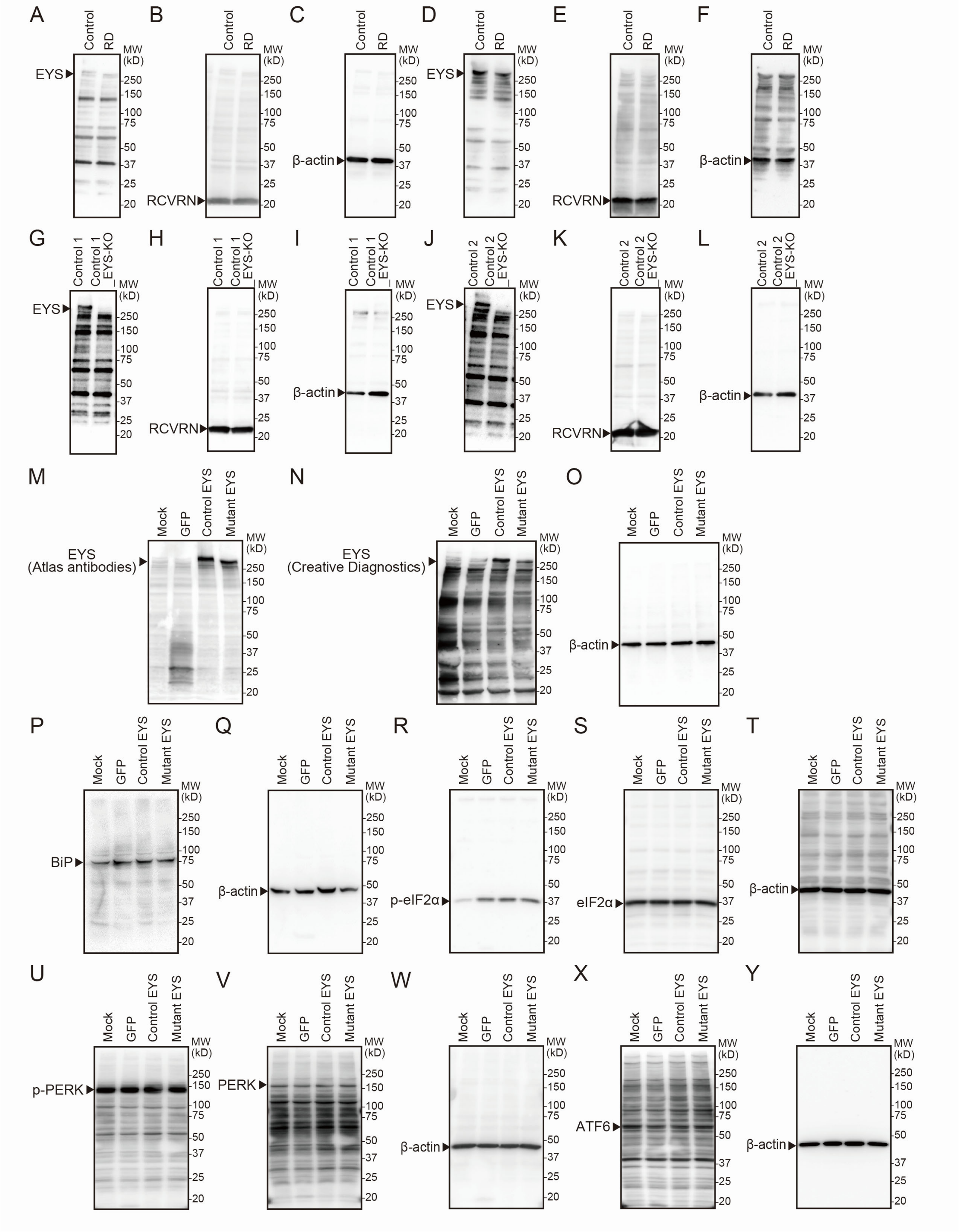
A
Control
RD
MW (kD)
EYS
250
150
100
75
50
37
25
20
B
RCVRN
C
β-actin
D
EYS
E
RCVRN
F
β-actin
G
Control 1
Control 1 EYS-KO
EYS
H
RCVRN
I
β-actin
J
Control 2
Control 2 EYS-KO
EYS
K
RCVRN
L
β-actin
M
Mock
GFP
Control EYS
Mutant EYS
EYS (Atlas antibodies)
N
EYS (Creative Diagnostics)
O
β-actin
P
BiP
Q
β-actin
R
p-eIF2α
S
eIF2α
T
β-actin
U
p-PERK
V
PERK
W
β-actin
X
ATF6
Y
β-actin

Figure S8. Full-length pictures of western blot analysis presented in the main and supplemental figures, Related to Figures 3, 4 and Supplemental Figure S4

(A) Full-length picture of the top left blot presented in Figure 3C.

(B) Full-length picture of the middle left blot presented in Figure 3C.

(C) Full-length picture of the bottom left blot presented in Figure 3C.

(D) Full-length picture of the top right blot presented in Figure 3C.

(E) Full-length picture of the middle right blot presented in Figure 3C.

(F) Full-length picture of the bottom right blot presented in Figure 3C.

(G) Full-length picture of the blot presented in Figure 4C.

(H) Full-length picture of the top blot presented in Figure 4C.

(I) Full-length picture of the bottom blot presented in Figure 4C.

(J) Full-length picture of the blot presented in Figure 4C.

(K) Full-length picture of the blot presented in Figure 4C.

(L) Full-length picture of the blot presented in Figure 4C.

(M) Full-length picture of the blot presented in Supplemental Figure 4C.

(N) Full-length picture of the blot presented in Supplemental Figure 4C.

(O) Full-length picture of the blot presented in Supplemental Figure 4C.

(P) Full-length picture of the blot presented in Supplemental Figure 4F.

(Q) Full-length picture of the blot presented in Supplemental Figure 4F.

(R) Full-length picture of the blot presented in Supplemental Figure 4F.

(S) Full-length picture of the blot presented in Supplemental Figure 4F.

(T) Full-length picture of the blot presented in Supplemental Figure 4F.

(U) Full-length picture of the blot presented in Supplemental Figure 4F.

(V) Full-length picture of the blot presented in Supplemental Figure 4F.

(W) Full-length picture of the blot presented in Supplemental Figure 4F.

(X) Full-length picture of the blot presented in Supplemental Figure 4F.

(Y) Full-length picture of the blot presented in Supplemental Figure 4F.

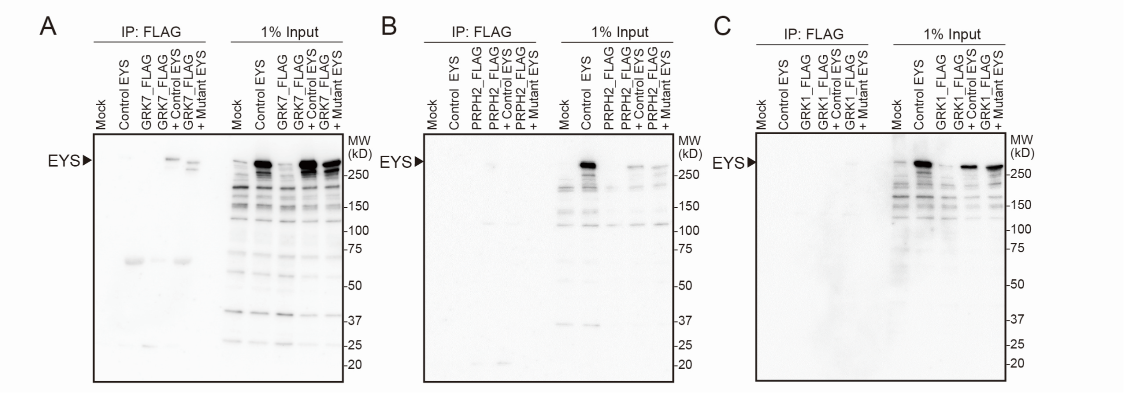

Figure S9. Full-length pictures of immunoprecipitation presented in the main and supplemental figures, Related to Figure 3 and Supplemental Figure 5

(A) Full-length picture of the top left blot presented in Figure 3H.

(B) Full-length picture of the top left blot presented in Figure Supplemental 5A.

(C) Full-length picture of the top left blot presented in Figure Supplemental 5E.

Table S1. List of human iPSCs

	iPSC name	Clone name	Age	Gender	Origin	Variant	Visual acuity (OD / OS)	Age of onset (Night blindness)
Healthy control 1	Clone 1	HPS 3920	49	M	PBMC	N/A	N/A	N/A
Healthy control 2	Clone 2	HPS 5234	64	M	PBMC	N/A	N/A	N/A
Patient 1	Clone 1	HPS 3933	84	M	Dermal fibroblast	c.8805C>A (homozygous)	HM / HM	33
Patient 2	Clone 2	HPS 3927	30	M	Dermal fibroblast	c.8805C>A (homozygous)	1.2 (20/17) / 1.5 (20/13)	20

PBMC: peripheral blood mononuclear cell, N/A: not applicable, OD: oculus dexter, OS: oculus sinister, HM: hand motion

Table S2. List of antibodies used for immunocytochemistry, immunohistochemistry and western blot (WB) analysis

Target	Host	Dilution	Source	Catalog Number
Acetylated-Tubulin	Mouse Monoclonal	1:3,000	Sigma Aldrich	T7451
Arl13b	Mouse Monoclonal	1:500	OriGene	75-287
Arrestin (SAG)	Mouse Monoclonal	1:1,000	Lifespan Biosciences	LS-B10410
Arrestin3	Mouse Monoclonal	1:500	Abcam	ab174435
ATF6	Mouse Monoclonal	1:500	Abcam	ab122897
β-actin	Mouse Monoclonal	1:10,000	Sigma-Aldrich	A5441
Cleaved Caspase-3	Rabbit Polyclonal	1:400	Cell Signaling Technology	#9661
eIF2α	Rabbit Polyclonal	1:1,000	Cell Signaling Technology	9722
EYS	Rabbit Polyclonal	1:1,000 (WB) 1:250	Atlas Antibodies	HPA027103
EYS	Mouse Monoclonal	1:1,000 (WB) 1:250	Creative diagnostics	CABT-B1316
GFP	Chicken Polyclonal	1:1,000	Abcam	ab13970
Gnat2	Rabbit Polyclonal	1:400	MBL International Corporation	PM075
GNAT2	Rabbit Polyclonal	1:1,000	Lifespan Biosciences	LS-C186359
GRK1	Mouse Monoclonal	1:500	Thermo Fisher Scientific	MA1-720
GRK7	Mouse Monoclonal	1:500	R & D Systems	MAB4700
GRK7	Rabbit Polyclonal	1:500	Atlas Antibodies	HPA075093
GRP78 BiP	Rabbit Polyclonal	1:1,000	Abcam	ab21685
IFT88	Rabbit Polyclonal	1:250	Proteintech	13967-1-AP
Nanog	Rabbit Polyclonal	1:500	REPROCELL	RCAB003P
Oct-4	Rabbit Polyclonal	1:500	Merck	AB3209
Opsin Blue	Rabbit Polyclonal	1:1,000	Merck	AB5407

Opsin Red/Green	Rabbit Polyclonal	1:500	Merck	AB5405
Peripherin-2	Mouse Monoclonal	1:2,000	Merck	MABN293
Peripherin-2	Rabbit Polyclonal	1:400	Abcam	ab122057
PERK	Rabbit Polyclonal	1:1,000	Cell Signaling Technology	3192
phospho-eIF2α	Rabbit Polyclonal	1:1,000	Cell Signaling Technology	3398
phospho-PERK	Rabbit Polyclonal	1:1,000	Cell Signaling Technology	3179
Recoverin	Rabbit Polyclonal	1:1,000	Proteintech	10073-1-AP
Opsin (Rhodopsin)	Mouse Monoclonal	1:1,000	Sigma Aldrich	O4886
SSEA4	Mouse Monoclonal	1:1,000	Merck	MAB4304
Tra1-60	Mouse Monoclonal	1:1,000	Cell Signaling Technology	#4746
Tom20	Rabbit Polyclonal	1:100	Santa Cruz Biotechnology	Sc11415

Supplemental Materials and Methods

Retinal organoid differentiation from human iPSCs

For producing retinal organoids, we employed the differentiation protocol described in previous reports (Figure 1A) (1). Human iPSC colonies on Day 7 were treated with 5 µM SB431542 (Cayman Chemical Company, Ann Arbor, MI) and 300 nM smoothened agonist (SAG; Enzo Life Sciences, New York, NY) for 24 hours prior to differentiation. On the following day, iPSCs were dissociated into single cells, plated in 96-well V-bottomed plates (Sumitomo Bakelite, Tokyo, Japan), and cultured in medium containing 45% Iscove's modified Dulbecco's medium (IMDM; Thermo Fisher Scientific), 45% Ham's F12 Nutrient Mix, Glutamax Supplement (Thermo Fisher Scientific), 1% chemically defined lipid concentrate (Thermo Fisher Scientific), 450 µM monothioglycerol (Sigma-Aldrich, St. Louis, MO), and 10% Knockout Serum Replacement (KSR; Thermo Fisher Scientific) in the presence of 20 µM Y-27632 and 30 nM SAG. On Day 3, recombinant human Bone Morphogenetic Protein 4 (BMP4; R&D Systems, Minneapolis, MN) was added to the medium at a final concentration of 1.5 nM. The medium was changed every three days until Day 18. The iPSC-derived organoids containing neural retina (NR) epithelium were subjected to two-step induction-reversal culture under the following conditions. Organoids on Day 18 were transferred from 96-well plates to 90 mm non-cell-adhesive petri dishes (PrimeSurface Schale 90; Sumitomo Bakelite) and further cultured for three days in suspension with DMEM/F12 - Glutamax medium (Thermo Fisher Scientific) containing 1% N2 supplement (Thermo Fisher Scientific), 3 µM CHIR99021 (GSK3-inhibitor; Cayman Chemical Company) and 5 µM SU5402 (FGFR-inhibitor; Sigma-Aldrich). Following this, the floating organoids were cultured in medium containing DMEM/F12 - Glutamax, 1% N2 supplement, 10% Fetal Bovine Serum (FBS; Thermo Fisher Scientific), 0.5 µM retinoic acid (RA) (Sigma-Aldrich), 0.1 mM taurine (Sigma-Aldrich) and 0.25 µg/ml Fungizone (Thermo Fisher) under 5% CO2 and 40% oxygen. The medium was changed twice a week until the end of each experiment. 100 U/mL penicillin and 100 µg/mL streptomycin (Thermo Fisher Scientific) were added to all media.

Retinal organoids that plunged to the following states during the differentiation process were not used for experiments or analysis: (i) Failure to form embryoid body (EB)-like aggregates, (ii) cystoid formation, (iii) disruption of organoids around Day 50-100, (iv) lack of brush-like structure on the surface by Day 180. The shape and size of typical retinal organoids is round and between approximately 1 and 3 mm. On Day 180, retinal organoids developing brush-like structure on more than one-fourth region of its surface were picked up and used for immunocytochemistry. To

evaluate protein localization in the OS region, we targeted areas where the OS marker Peripherin2 was stained. Regarding quantification of immunocytochemistry, three images were randomly acquired in targeted areas per organoid. Three or six organoids of each clone were analyzed. For western blot and ELISA analysis, only organoids that formed a brush-like structure all around were collected, in order to match the amount of targeted molecule photoreceptor cells as much as possible. Three organoids of similar size were pick up from the same batch and compared.

Single-cell RNA sequencing

On Day 70, three retinal organoids were collected and dissociated using Accumax Cell/Tissue Dissociation Solution (Lagen Laboratories, Rochester, MI) at 37°C for 10 min. The dissociated cells were suspended in PBS containing 10 μM Y-27632 and 0.1% Bovine serum albumin (BSA), which was then immediately followed by a library preparation targeting single cells using Chromium Next GEM Single Cell 3' Reagent Kits v3.1 (10×Genomics, CA) according to the manufacturer's instructions. Six thousand cells were analyzed, and the library was sequenced on DNB Seq-G400 (MGI Tech, Shenzhen, China). Alignment to the human reference genome GRCh38 and UMI counting were conducted by Cell Ranger v6.1.2 pipeline (10x Genomics). Data analyses were implemented by the Seurat package v4.0.5.

Immunocytochemistry with adherent cells or retinal organoids

Two-dimensional adherent cells were fixed in 4% paraformaldehyde (PFA; Nacalai-Tesque) for 15 min at room temperature, washed with PBS and permeabilized in PBS-T (PBS with 0.1% Tween 20) containing 0.2% Triton X-100 (Nacalai-Tesque) for 30 min at room temperature, and then incubated with blocking buffer (10% Normal Goat serum (Vector Laboratories, Burlingame, CA) in PBS-T). After incubation with primary antibodies overnight at 4°C, cells were washed three times with PBS and incubated with appropriate secondary antibodies for one hour at room temperature.

The immunostaining procedure for retinal organoids was performed by the following steps. Organoids were fixed with 4% PFA for 20 min at room temperature. They were immediately washed with PBS before incubated in 30% sucrose in PBS at 4°C overnight for cryo-protection. The fixed organoids were embedded in OCT compound (Sakura Finetek, Tokyo, Japan) and stored at -80°C. Cryo-sections of 12 μm thickness were made with a Cryostat (Leica, Wetzlar, Germany). Samples were permeabilized with 0.3% Triton X-100 in PBS-T for 15 min. Slides were encircled with a hydrophobic pen (Vector Laboratories) and incubated with blocking buffer for one hour at room temperature. They were incubated with primary antibodies dilated in blocking buffer at 4°C overnight,

followed by incubation with secondary antibodies conjugated with Alexa 488, 594 and DAPI at room temperature for one hour. The primary antibodies used in this assay are listed in Supplemental Table 2.

Cell images were acquired with FLUOVIEW FV3000 (Olympus Corporation, Tokyo, Japan). To evaluate the colocalization of immunofluorescence, Z-stack images were acquired and used in orthogonal projections. For quantifying the data, acquired images were transformed and analyzed with IN CELL Developer toolbox software 1.9.

Generation of *EYS* knockout iPSCs with CRISPR/Cas9 technology

EYS knockout (*EYS*-KO) iPSCs were produced by CRISPR/Cas9 gene editing technology using a method described previously (2). Guide RNAs were designed to target exon 4 of *EYS* gene (see Figure 4A for sequences). 5×10^5 control iPSCs were electroporated with single-guide RNA and Cas9 protein using Amaxa P4 Primary Cell 4D-Nucleofector™ X Kit (Lonza, Basel, Switzerland) and 4D-Nucleofector (Lonza). The increased iPSCs were plated at a low density in 10 cm dishes and cultured for 9 days until iPSC colonies were ready to be picked up. iPSC clones were manually isolated, and each clone was analyzed by Sanger sequencing to confirm *EYS* gene disruption.

Generation of *eys*-knockout zebrafish with CRISPR/Cas9 technology

For the generation of *eys*-knockout (*eys*-KO) fish, we used clustered regularly interspaced short palindromic repeats (CRISPR)/Cas9 method. crRNA targeting the coding region of exon 2 of *eys* gene (GCTGCAGGTGTTCTCCATGAggg) (3), where the protospacer adjacent motifs (PAMs) are indicated by lower cases, was synthesized (Alt-R™ CRISPR-Cas9 System; Integrated DNA Technologies, Coralville, IA). The crRNA:tracrRNA duplex (3 fmol) and hSpCas9 mRNA (300 pg) were injected into zebrafish embryos at the 1-cell stage. hSpCas9 was in vitro-transcribed with mMESSAGE mMACHINE Kit (Thermo Fisher Scientific) by using pCS2+ hSpCas9 plasmid as a template (a gift from Masato Kinoshita; Addgene plasmid # 51815). The frame-shift mutation was identified by analyzing the PCR products of the targeted *eys* locus amplified by the primer pair (eys-56f: GCCCTGTGTACAGCCAGGTAAC and eys-284r: GAAACAAACAAGAGGTCTCCAAGT) with Heteroduplex Mobility Assay followed by DNA sequencing.

Light or dark adaptation, Immunohistochemistry and Hematoxylin-Eosin staining with zebrafish retina

Zebrafish at 3 or 6 months postfertilization (mpf) were used. Zebrafish at 3 mpf were incubated in darkness for 6 hours or exposed to white LED light (approximately 5,000-7,000 lx) for 15 min before sacrifice. They were fixed with 4% PFA in PBS at 4°C overnight. Their eyes were isolated and cryoprotected in 30% sucrose overnight. They were embedded in OCT compound and stocked at -80°C before sectioning. Sectioning of 10 μm thickness was conducted with a Cryostat. Immunostaining and observation process was carried out as well as retinal organoids. Some sections were stained with hematoxylin and eosin (MUTO PURE CHEMICALS, Tokyo, Japan). Terminal deoxynucleotidyl transferase dUTP nick end labeling (TUNEL) was performed with ApopTag Fluorescein In Situ Apoptosis Detection Kit (Merck, Burlington, MA) according to the manufacturer's suggestions. The number of TUNEL-positive cells in the entire photoreceptor cell layer of each zebrafish were counted manually. For DAPI counting, sections sliced at the optic nerve were immunostained. Images were acquired with FV3000 and analyzed with ImageJ software. The number of rod or cone nuclei in a range of 100 μm was counted, 150 μm or 300 μm distant from the center of the optic nerve.

Immunohistochemistry with paraffin sections of pig retina

Paraffin sections of the pig retina were deparaffinized by the following processes. Slides were incubated in Xylene (Nacalai-Tesque) for 5 min three times, in 100% ethanol for 3 min twice, and in 90%, 70% and 50% ethanol for 3 min each. The sections were washed with water and boiled in a citrate antigen retrieval buffer (10 mM Sodium Citrate, 0.05% Tween 20, pH 6.0) at 121°C for 5 min. Slides were cooled in PBS and were encircled with a hydrophobic pen. The sections were permeabilized with 0.3% Triton X-100 in PBS-T for 30 min. Immunofluorescence was conducted in the same way as for retinal organoids. For diaminobenzidine (DAB) staining, Avidin blocking (Vector Laboratories) was added to blocking buffer (1 drop / 250μl) and, slides were incubated for one hour at room temperature. Primary antibodies were dilated in blocking buffer with added Biotin blocking (Vector Laboratories; 1 drop / 250μl). Samples were incubated with primary antibodies at 4°C overnight. On the following day, they were rinsed three times with PBS-T. Peroxide Block with 0.3% H_2O_2 (Nacalai-Tesque) in PBS was applied for 30 min to block endogenous peroxide activity. They were washed with PBS-T three times, followed by incubation with secondary antibodies conjugated with biotin (Vector Laboratories) at room temperature for 30 min. Sections were then rinsed three times in PBS-T and incubated in Avidin-Biotin complex (Vector Laboratories) for 30 min. They were washed with PBS-T three times before detection with DAB + Substrate Chromogen system (Agilent Technologies, Santa Clara, CA). They were rinsed with PBS three times and

counterstained with Hematoxylin for 5 min. The sections were then rinsed in water for 10 min before the dehydration process. Each slide was dipped in 50%, 70%, 90% and 100% ethanol for 3 min successively and in Xylene for 5 min three times. Finally, the slides were mounted under glass coverslips using Mount quick (Daido Sangyo, Toda, Saitama, Japan). Images were captured by BX51 Discussion Microscope (Olympus Corporation).

Scanning electron microscopy, transmission electron microscopy and immunoelectron microscopy

Retinal organoids on Day 180 were fixed in PBS containing 2% glutaraldehyde (FUJIFILM Wako Pure Chemical Corporation, Osaka, Japan) and 4% PFA at 4°C overnight. The sections were placed for 90 min in 1% OsO_4 in 0.1 M PB. Following dehydration in a series of graded concentrations of ethanol (50%, 60%, 70%, 80%, 90%, 99%, and 100%) and propylene oxide, the fixed cell blocks were embedded in epoxy resin (Luveak 812; Nacalai-Tesque). Ultrathin sections (100 nm thickness) were prepared on an ultramicrotome (ARTOS 3D; Leica) equipped with a diamond knife (SYM jumbo, 45 degrees; SYNTEK, Yokohama, Japan). Serial sections were collected on a cleaned silicon wafer strip held by a micromanipulator (MN-153; NARISHIGE, Tokyo, Japan) for backscattered electron microscopy. Those sections were stained at room temperature using 2% aqueous uranyl acetate (20 min) and Reynolds' lead citrate (3 min). The sections were imaged using a scanning electron microscope (JSM-7900F; JEOL, Tokyo, Japan) supported by software (Array Tomography Supporter (version 1.0.0.0); System in Frontier, Tokyo, Japan) that enables automated imaging. Regarding zebrafish samples, cryo-sections of 10 μm thickness made with a Cryostat were used for transmission electron microscopy experiments. The procedures were the same as for organoids except for placing in 1% OsO_4 for 60 min. The sections were examined with an electron microscope (JEM-1400; JEOL).

Immunoelectron microscopy was conducted by the following procedures. The sections were washed with PBS for 10 min before incubation for 30 min with PBS containing 10% goat serum and 0.05% Photoflo (Kodak, Rochester, NY). They were incubated with anti-EYS antibody (1:250) overnight at 4°C. After rinsing with PBS, the sections were incubated with 1:100-diluted Nanogold anti-rabbit IgG (Nanoprobes, Yaphank, NY) overnight at 4°C. They were rinsed with PBS and 0.1 M phosphate buffer (PB) before being incubated with 1% glutaraldehyde for 10 min. After rinsing with PB and water, immunoreactivities were developed in the dark using Silver Enhancement kit, HQ silver (Nanoprobes). After washing, the sections were placed in 1% $OsO_4$for 60 min, dehydrated in an ethanol series, and flat-embedded in epoxy-resin. These experiments were performed at the

Division of Electron Microscopic Study, Center for Anatomical Studies, Graduate School of Medicine, Kyoto University.

Plasmid construction and transfection to 293T cells

293T cells established in RIKEN BRC CELL BANK (Resource No. RCB2202) were used in the current study. Human GRK7 cDNA was obtained from DANAFORM (Yokohama, Japan). Human GRK1 and PRPH2 cDNA was purchased from Horizon Discovery (Cambridge, UK). EYS cDNA was extracted from human iPSCs and synthesized as three divided fragments before combining for full length. Each cDNA fragment was inserted into pcDNA3.1$^{(+)}$ (Thermo Fisher Scientific). Cells were transfected with Lipofectamine 2000 (Thermo Fisher Scientific). DNA plasmid of 4 μg and 10 μl of Lipofectamine 2000 were diluted in 250 μl Opti-MEM (Thermo Fisher Scientific) and incubated for 20 min. A density of 293T cells collected in Opti-MEM were adjusted to 1.5×10^5 cells per 250 μl. Diluted DNA/Lipofectamine solution was added to the cells and plated into 6-well plates. About 24 hours later, the DNA/Lipofectamine mixture was removed by changing the media. Thirty-six hours after Lipofection, GFP fluorescence was observed with a fluorescence microscope (Olympus Corporation), and cells were collected for the following experiment.

Construction, preparation and infection of helper-dependent adenoviral vector (HDAdV)

To generate the human *EYS* gene expression vector, a plasmid DNA encoding the full-length human EYS cDNA, driven by the human cytomegalovirus (CMV) enhancer/promoter, was constructed with the IRES-linked enhanced green fluorescent protein (EGFP). Subsequently, the EYS-IRES-EGFP cassette was subcloned into the HDAdV transfer plasmid between the left and the right end 400 bp, containing the inverted terminal repeats (ITR) and the packaging signal, of human adenovirus serotype 5. This transfer plasmid also encodes non-viral stuffer DNA to adjust the size of HDAdV genome. The HDAdV was propagated by serial passages on the 116 cells with addition of AdNG163R-2 helper virus and purified, as described previously (4). Vector genome titer was determined by quantitative real-time PCR analysis. For the infection to retinal organoids, the HDAdV was prepared to a final titer 2.0×10^{10} vector genome copy (gc)/mL. On day 200, each organoid was transferred to the well of 96-well plate and incubated with the vector in 100 μL media for 36 hours. Infected organoids were then, picked up again to 90 mm dishes with fresh media. Half media was changed every three or four days. Seven days after removing the virus, samples were collected and used for experiments.

Western blot analysis

Cells were harvested and dissolved in RIPA buffer (Nacalai-Tesque) containing protease inhibitor (Sigma-Aldrich) and phosphatase inhibitor (Sigma-Aldrich) for 30 min before being centrifuged at 15,000 × g for 15 min at 4°C. Protein concentrations in the supernatants were determined using the bicinchoninic acid (BCA) assay kit (Thermo Fisher Scientific). Total protein extracts (10 µg per lane) were separated on 4-20% gradient polyacrylamide gels (Bio-Rad Laboratories, Hercules, CA) and transferred to an Immobilon-P membrane (Merck). Membranes were blocked with PVDF Blocking Reagent for Can Get Signal (TOYOBO, Osaka, Japan), hybridized with appropriate antibodies. Secondary antibody coupled with horseradish peroxidase and the ECL Prime detection kit (GE Healthcare, Chicago, IL) were used for detection. Images were acquired on ImageQuant LAS 4000 (GE Healthcare).

Immunoprecipitation with 293T cells

For co-transfection of EYS and GRK7 (or GRK1 or Peripherin-2) with FLAG, 2:1 mixed plasmid was transfected to 293T cells with Lipofectamine 3000 (Thermo Fisher Scientific). The protocol for using Lipofectamine 3000 was the same as for Lipofectamine 2000. The medium was changed 24 hours later, and cells were collected 36 hours after Lipofection. Transfected 293T cells were lysed on ice with buffer (50 mM Tris HCl, pH 7.6, with 150 mM NaCl, 1 mM EDTA, and 1% Triton X-100) containing protease and phosphatase inhibitor. Lysates were centrifuged (15,000 × g) for 15 min. Anti-FLAG Magnetic Beads (Sigma Aldrich) were used for FLAG IP according to the manufacturer's instructions. Briefly, 50 µl beads per sample were resuspended with TBS buffer (50 mM Tris HCl, 150 mM NaCl, pH 7.4). Protein extract of 3 mg was incubated with the equilibrated beads at 4°C overnight. The FLAG proteins were eluted from the magnetic beads by acid conditions with 500 µl of 0.1 M glycine HCl buffer, pH 3.0, and neutralized pH with 0.5 M Tris HCl, pH 8, with 1.5 M NaCl. The eluted solution was concentrated with Amicon Ultra (10 kD; Merck) and separated on 4-20% gradient polyacrylamide gels. It was transferred to an Immobilon P Membrane and blocked with PVDF Blocking Reagent for Can Get Signal. The following primary antibodies were used: EYS (1:1,000), FLAG (1:1,000, Cell Signaling Technology, Danvers, MA). Quantification of bands was conducted with Image J software.

Enzyme-linked immunosorbent Assay (ELISA)

Three organoids of similar sizes were prepared from the same batch. They were washed with PBS and incubated in 0.1M HCl before being homogenized. The samples were centrifuged at 1,000 × g

for 10 min, and supernatants were collected. Protein concentrations were determined by BCA assay kit to match protein levels among samples. cGMP levels in retinal organoids were measured by ELISA using a cGMP direct immunoassay kit (Cayman Chemical Company) following the manufacturer's instructions. Absorbance at 405 nm was measured using the VersaMax™ Microplate Reader (Molecular Devices, San Jose, CA). The results shown are an average of three or six independent experiments.

Light-induced damage analysis with retinal organoids

On Day 178, 9-cis-Retinal vitamin A analog (Cayman Chemical Company) was added to the media at a final concentration of 10 μM. After overnight incubation, Retinal organoids were picked up into new dishes with DMEM/F12, no phenol red (Thermo Fisher) to prevent nutrients from degrading, supplemented with 1% N2 supplement, 10% FBS, 0.5 μM RA, 0.1 mM taurine and 10 μM 9-cis-Retinal. The organoids were exposed to white, blue, green or red LED light, respectively, for 24 hours under 5% CO_2 at 37°C. The illuminance and intensity of LED lights were measured with a spectrophotometer C-7000 (SEKONIC, Tokyo, Japan). White light was 10,000 lx, and the other three colors were adjusted to 1,000 lx or 15 mW/cm^2. Dark control organoids were all from the same stock and were cultured simultaneously in another incubator. We confirmed by thermometer measurements that medium temperature was not affected by LED. Following light exposure, organoids were collected for further experiments. For measurement of reactive oxygen species (ROS), organoids were collected to the media containing CellROX Orange Reagent (Thermo Fisher) at a final concentration of 1 μM. A nuclear counterstain was conducted with 1 μg/ml Hoechst33342 (Thermo Fisher) at the same time. Organoids were incubated for 30 min at 37°C and washed three times with PBS. Fluorescence was observed with FV3000.

Reference

1. Kuwahara A, et al. Preconditioning the Initial State of Feeder-free Human Pluripotent Stem Cells Promotes Self-formation of Three-dimensional Retinal Tissue. *Sci Rep*. 2019;9(1):18936.

2. Howden SE, et al. Simultaneous Reprogramming and Gene Correction of Patient Fibroblasts. *Stem Cell Reports*. 2015;5(6):1109–1118.

3. Yu M, et al. Eyes shut homolog is required for maintaining the ciliary pocket and survival of photoreceptors in zebrafish. *Biol Open*. 2016;5(11):1662–1673.

4. Palmer DJ, Ng P. Physical and infectious titers of helper-dependent adenoviral vectors: a method of direct comparison to the adenovirus reference material. *Molecular Therapy*. 2004;10(4):792–798.
